# Supplementary material for: Directed Evolution of AAV Serotype 5 for Increased Hepatocyte Transduction and Retained Low Humoral Seroreactivity
Source: Mol Ther Methods Clin Dev. 2020 Oct 20;20:122–32. doi: 10.1016/j.omtm.2020.10.010 (PMC7773954; doi:10.1016/j.omtm.2020.10.010)
Supplement: Document S1. Figures S1–S6, Table S1, and Supplemental Methods [file mmc1.pdf]

OMTM, Volume ■ ■ ■

Supplemental Information

Directed Evolution of AAV Serotype 5 for Increased Hepatocyte Transduction and Retained Low Humoral Seroreactivity

Randolph Qian, Bin Xiao, Juan Li, and Xiao Xiao

Supplemental Figures

>MV1

MSFVDHPPDWLEEVGEGLREFLGLEAGPPKPKPNQQHQDQARGLVLPGYNYLGPGNGLDRGEP
VNRADEVAREHDISYNEQLEAGDNPYLKYNHADAEFQEKLADDTSFGGNLGKAVFQAKKRVLE
PFGLVEEGAKTAPTGKRIDDHFPKRKKARTEEDSKPSTSSDAEAGPSGSQQLQIPAQPASSLGADT
MSAGGGGPLGDNNQGADGVGNASGDWHCDSTWMGDRVVTKSTRTWVLPSYNNHQYREIKSG
SVDRSNANAYFGYSTPWGYFDFNRFHSHWSPRDWQRLINNYWGFRPRSLRVKIFNIQVKEVTVQ
DSTTTIANNLTSTVQVFTDDDYQLPYVVGNGTEGCLPAFPPQVFTLPQYGYATLNRDNTENPTER
SSFFCLEYFPSKMLRTGNNFEFTYNFEEVPFHSSLAPSQNLFKLANPLVDQYLYRFVSTNNTGGV
QFNKNLAGRYANTYKNWFPGPMGRTQGWNLGSGVNRASVSAFATTNRMELEGASYQVPPQPN
GMTNNLQGSNTYALENTMIFNSQPANPGTTATYLEGNMLITSESETQPVNRVAYNVGGQMATN
NQSSTTAPATGTYNLQEIVPGSVWMERDVYLQGPIWAKIPETGAHFHPSPAMGGFGLKHPPPMM
LIKNTPVPGNITSFSDVPVSSFITQYSTGQVTVEMEWELKKENSKRWNPEIQYTNNYNDPQFVDF
APDSTGEYRSTRPIGTRYLTRPL

>MV18

MSFVDHPPDWLEEVGEGLREFLGLEAGPPKPKPNQQHQDQARGLVLPGYNYLGPGNGLDRGEP
VNRADEVAREHDISYNEQLEAGDNPYLKYNHADAEFQEKLADDTSFGGNLGKAVFQAKKRVLE
PFGLVEEGAKTAPTGKRIDDHFPKRKKARTEEDSKPSTSSDAEAGPSGSQQLQIPAQPASSLGADT
MSAGGGGPLGDNNQGADGVGNASGDWHCDSTWMGDRVVTKSTRTWVLPSYNNHQYREIKSG
SVDRSNANAYFGYSTPWGYFDFNRFHSHWSPRDWQRLINNYWGFRPRSLRVKIFNIQVKEVTVQ
DSTTTIANNLTSTVQVFTDDDYQLPYVVGNGTEGCLPAFPPQVFTLPQYGYATLNRDNTENPTER
SSFFCLEYFPSKMLRTGNNFEFTYNFEEVPFHSSFAPSQNLFKLANPLVDQYLYRFVSTNNTGGV
QFNKNLAGRYANTYKNWFPGPMGRTQGWNLGSGVNRASVSAFATTNRMELEGASYQVPPQPN
GMTNNLQGSNTYALENTMIFNSQPANPGTTATYLEGNMLITSESETQPVNRVAYNVGGQMATN
NQSSTTAPATGTYNLQEIVPGSVWMERDVYLQGPIWAKIPETGAHFHPSPAMGGFGLKHPPPMM
LIKNTPVPGNITSFSDVPVSSFITQYSTGQVTVEMEWELKKENSKRWNPEIQYTNNYNDPQFVDF
APDSTGEYRTTRPIGTRYLTRPL

>MV20

MSFVDHPPDWLEEVGEGLREFLGLEAGPPKPKPNQQHQDQARGLVLPGYNYLGPGNGLDRGEP
VNRADEVAREHDISYNEQLEAGDNPYLKYNHADAEFQEKLADDTSFGGNLGKAVFQAKKRVLE
PFGLVEEGAKTAPTGKRIDDHFPKRKKARTEEDSKPSTSSDAEAGPSGSQQLQIPAQPASSLGADT
MSAGGGGSLGDNNQGADGVGNASGDWHCDSTWMGDRVVTKSTRTWVLPSYNNHQYREIKSG
SVDGSNANAYFGYSTPWGYFDFNRFHSHWSPRDWQRLINNYWGFRPRSLRVKIFNIQVKEVTV
QDSTTTIANNLTSTVQVFTDDDYQLPYVVGNGTEGCLPAFPPQVFTLPQYGYATLNRDNTENPTE
RSSFFCLEYFPSKMLRTGNNFEFTYNFEEVPFHSSFAPSQNLFKLANPLVDQYLYRFVSTNNTGG
VQFNKNLAGRYANTYKNWFPGPIGRTQGWNLGSGVNRASVSAFATTNRMELEGASYQVPPQPN
GMTNNLQGSNTYALENTMIFNSQPANPGTTATYLEGNMLITSESETQPVNRVAYNVGGQMATN
NQSSTTTPATGTYNLQEIVPGSVWMERDVYLQGPIWAKIPETGAHFHPSPAMGGFGLKHPPPMM
LIKNTPVPGNITSFSDVPVSSFITQYSTGQVTVEMEWELKKENSKRWNPEIQYTNNYNDPQFVDF
APDSTGEYRSTRPIGTRYLTRPL

>MV50

MSFVDHPPDWLEEVGEGLREFLGLEAGPPKPKPNQQHQDQARGLVLPGYNYLGPGNGLDRGEP
VNRADEVAREHDISYNEQLEAGDNPYLKYNHADAEFQEKLADDTSFGGNLGKAVFQAKKRVLE
PFGLVEEGAKTAPTGKRIDDHFPKRKKARTEEDSKPSTSSDAEAGPSGSQQLQIPAQPASSLGADT
MSAGGGGPLGDNNQGADGVGNASGDWHCDSTWMGDRVVTKSTRTWVLPSYNNHQYREIKSG
SVDRSNANAYFGYSTPWGYFDFNRFHSHWSPRDWQRLINNYWGFRPRSLRVKIFNIQVKEVTVQ
DSTTTIANNLTSTVQVFTDDDYQLPYVVGNGTEGCLPAFPPQVFTLPQYGYATLNRDNTENPTER
SSFFCLEYFPSKMLRTGNNFEFTYNFEEVPFHSSFAPSQNLFKLANPLVDQYLYRFVSTNNTGGV
QFNKNLAGRYANTYKNWFPGPMGRTQGWNLGSGVNRASVSAFATTNRMELEGASYQVPPQPN
GMTNNLQGSNTYALENTMIFNSQPANPGTTATYLEGNMLITSESETQPVNRVAYNVGGQMATN
NQSSTTAPATGTYNLQEIVPGSVWMERDVYLQGPIWAKIPETGAHFHPSPAMGGFGLKHPPPMM
LIKNTPVPGNITSFSDVPVSSFITQYSTGQVTVEMEWELKKENSKRWNPEIQYTNNYNDPQFVDF
APDGTGEYRSTRPIGTRYLTRPL

>MV53

MSFVDHPPDWLEEVGEGLREFLGLEAGPPKPKPNQQHQDQARGLVLPGYNYLGPGNGLDRGEP
VNRADEVAREHDISYNEQLEAGDNPYLKYNHADAEFQEKLADDTSFGGNLGKAVFQAKKRVLE
PFGLVEEGAKTAPTGKRIDDHFPKRKKARTEEDSKPSTSSDAEAGPSGSQQLRIPAQPASSLGADT
MSAGGGGPLGDNNQGADGVGNASGDWHCDSTWMGDRVVTKSTRTWVLPSYNNHQYREIKSG
SVDRSNANAYFGYSTPWGYFDFNRFHSHWSPRDWQRLINNYWGFRPRSLRVKIFNIQVKEVTVQ
DSTTTIANNLTSTVQVFTDDDYQLPYVVGNGTEGCLPAFPPQVFTLPQYGYATLNRDNTENPTER
SSFFCLEYFPSKMLRTGNNFEFTYNFEEVPFHSSFAPSQNLFKLANPLVDQYLYRFVSTNNTGGV
QFNKNLAGRYANTYKNWFPGPMGRTQGWNLGSGVNRASVSAFATTNRMELEGASYQVPPQPN
GMTNNLQGSNTYALENTMIFNSQPANPGTTATYLEGNMLITSESETQPVNRVAYNVGGQMATN
NQSSTTAPATGTYNLQEIVPGSVWMERDVYLQGPIWAKIPETGAHFHPSPAMGGFGLKHPPPMM
LIKNTPVPGNITSFSDVPVSSFITQYSTGQVTVEMEWELKKENSKRWNPEIQYTNNYNDPQFVDF
APDSTGEYRTTRPIGTRYLTRPL

Figure S1: Amino Acid Sequence for Best Performing AAV5 Variants

Amino acid sequences for best performing variants. The mutations that differ from AAV5 are highlighted in red.

```
AAV5    1 MSFVDHPPDWLEEVGEGLREFLGLEAGPPKPKPNQQHQDQARGLVLPGYNYLGPGNGLDR
MV1     1 MSFVDHPPDWLEEVGEGLREFLGLEAGPPKPKPNQQHQDQARGLVLPGYNYLGPGNGLDR
MV18    1 MSFVDHPPDWLEEVGEGLREFLGLEAGPPKPKPNQQHQDQARGLVLPGYNYLGPGNGLDR
MV20    1 MSFVDHPPDWLEEVGEGLREFLGLEAGPPKPKPNQQHQDQARGLVLPGYNYLGPGNGLDR
MV50    1 MSFVDHPPDWLEEVGEGLREFLGLEAGPPKPKPNQQHQDQARGLVLPGYNYLGPGNGLDR
MV53    1 MSFVDHPPDWLEEVGEGLREFLGLEAGPPKPKPNQQHQDQARGLVLPGYNYLGPGNGLDR

AAV5   61 GEPVNRADEVAREHDISYNEQLEAGDNPYLKYNHADAEFQEKLADDTSFGGNLGKAVFQA
MV1    61 GEPVNRADEVAREHDISYNEQLEAGDNPYLKYNHADAEFQEKLADDTSFGGNLGKAVFQA
MV18   61 GEPVNRADEVAREHDISYNEQLEAGDNPYLKYNHADAEFQEKLADDTSFGGNLGKAVFQA
MV20   61 GEPVNRADEVAREHDISYNEQLEAGDNPYLKYNHADAEFQEKLADDTSFGGNLGKAVFQA
MV50   61 GEPVNRADEVAREHDISYNEQLEAGDNPYLKYNHADAEFQEKLADDTSFGGNLGKAVFQA
MV53   61 GEPVNRADEVAREHDISYNEQLEAGDNPYLKYNHADAEFQEKLADDTSFGGNLGKAVFQA

AAV5  121 KKRVLEPFGLVEEGAKTAPTGKRIDDHFPKRKKARTEEDSKPSTSSDAEAGPSGSQQLQI
MV1   121 KKRVLEPFGLVEEGAKTAPTGKRIDDHFPKRKKARTEEDSKPSTSSDAEAGPSGSQQLQI
MV18  121 KKRVLEPFGLVEEGAKTAPTGKRIDDHFPKRKKARTEEDSKPSTSSDAEAGPSGSQQLQI
MV20  121 KKRVLEPFGLVEEGAKTAPTGKRIDDHFPKRKKARTEEDSKPSTSSDAEAGPSGSQQLQI
MV50  121 KKRVLEPFGLVEEGAKTAPTGKRIDDHFPKRKKARTEEDSKPSTSSDAEAGPSGSQQLQI
MV53  121 KKRVLEPFGLVEEGAKTAPTGKRIDDHFPKRKKARTEEDSKPSTSSDAEAGPSGSQQLRI

AAV5  181 PAQPASSLGADTMSAGGGGPLGDNNQGADGVGNASGDWHCDSTWMGDRVVTKSTRTWVLP
MV1   181 PAQPASSLGADTMSAGGGGPLGDNNQGADGVGNASGDWHCDSTWMGDRVVTKSTRTWVLP
MV18  181 PAQPASSLGADTMSAGGGGPLGDNNQGADGVGNASGDWHCDSTWMGDRVVTKSTRTWVLP
MV20  181 PAQPASSLGADTMSAGGGGSLGDNNQGADGVGNASGDWHCDSTWMGDRVVTKSTRTWVLP
MV50  181 PAQPASSLGADTMSAGGGGPLGDNNQGADGVGNASGDWHCDSTWMGDRVVTKSTRTWVLP
MV53  181 PAQPASSLGADTMSAGGGGPLGDNNQGADGVGNASGDWHCDSTWMGDRVVTKSTRTWVLP

AAV5  241 SYNNHQYREIKSGSVDGSNANAYFGYSTPWGYFDFNRFHSHWSPRDWQRLINNYWGFRPR
MV1   241 SYNNHQYREIKSGSVDRSNANAYFGYSTPWGYFDFNRFHSHWSPRDWQRLINNYWGFRPR
MV18  241 SYNNHQYREIKSGSVDRSNANAYFGYSTPWGYFDFNRFHSHWSPRDWQRLINNYWGFRPR
MV20  241 SYNNHQYREIKSGSVDGSNANAYFGYSTPWGYFDFNRFHSHWSPRDWQRLINNYWGFRPR
MV50  241 SYNNHQYREIKSGSVDRSNANAYFGYSTPWGYFDFNRFHSHWSPRDWQRLINNYWGFRPR
MV53  241 SYNNHQYREIKSGSVDRSNANAYFGYSTPWGYFDFNRFHSHWSPRDWQRLINNYWGFRPR

AAV5  301 SLRVKIFNIQVKEVTVQDSTTTIANNLTSTVQVFTDDDYQLPYVVGNGTEGCLPAFPPQV
MV1   301 SLRVKIFNIQVKEVTVQDSTTTIANNLTSTVQVFTDDDYQLPYVVGNGTEGCLPAFPPQV
MV18  301 SLRVKIFNIQVKEVTVQDSTTTIANNLTSTVQVFTDDDYQLPYVVGNGTEGCLPAFPPQV
MV20  301 SLRVKIFNIQVKEVTVQDSTTTIANNLTSTVQVFTDDDYQLPYVVGNGTEGCLPAFPPQV
MV50  301 SLRVKIFNIQVKEVTVQDSTTTIANNLTSTVQVFTDDDYQLPYVVGNGTEGCLPAFPPQV
MV53  301 SLRVKIFNIQVKEVTVQDSTTTIANNLTSTVQVFTDDDYQLPYVVGNGTEGCLPAFPPQV

AAV5  361 FTLPQYGYATLNRDNTENPTERSSFFCLEYFPSKMLRTGNNFEFTYNFEEVPFHSSFAPS
MV1   361 FTLPQYGYATLNRDNTENPTERSSFFCLEYFPSKMLRTGNNFEFTYNFEEVPFHSSLAPS
MV18  361 FTLPQYGYATLNRDNTENPTERSSFFCLEYFPSKMLRTGNNFEFTYNFEEVPFHSSFAPS
MV20  361 FTLPQYGYATLNRDNTENPTERSSFFCLEYFPSKMLRTGNNFEFTYNFEEVPFHSSFAPS
MV50  361 FTLPQYGYATLNRDNTENPTERSSFFCLEYFPSKMLRTGNNFEFTYNFEEVPFHSSFAPS
MV53  361 FTLPQYGYATLNRDNTENPTERSSFFCLEYFPSKMLRTGNNFEFTYNFEEVPFHSSFAPS

AAV5  421 QNLFKLANPLVDQYLYRFVSTNNTGGVQFNKNLAGRYANTYKNWFPGPMGRTQGWNLGSG
```

```
MV1   421 QNLFKLANPLVDQYLYRFVSTNNTGGVQFNKNLAGRYANTYKNWFPGPMGRTQGWNLGSG
MV18  421 QNLFKLANPLVDQYLYRFVSTNNTGGVQFNKNLAGRYANTYKNWFPGPMGRTQGWNLGSG
MV20  421 QNLFKLANPLVDQYLYRFVSTNNTGGVQFNKNLAGRYANTYKNWFPGPIGRTQGWNLGSG
MV50  421 QNLFKLANPLVDQYLYRFVSTNNTGGVQFNKNLAGRYANTYKNWFPGPMGRTQGWNLGSG
MV53  421 QNLFKLANPLVDQYLYRFVSTNNTGGVQFNKNLAGRYANTYKNWFPGPMGRTQGWNLGSG

AAV5  481 VNRASVSAFATTNRMELEGASYQVPPQPNGMTNNLQGSNTYALENTMIFNSQPANPGTTA
MV1   481 VNRASVSAFATTNRMELEGASYQVPPQPNGMTNNLQGSNTYALENTMIFNSQPANPGTTA
MV18  481 VNRASVSAFATTNRMELEGASYQVPPQPNGMTNNLQGSNTYALENTMIFNSQPANPGTTA
MV20  481 VNRASVSAFATTNRMELEGASYQVPPQPNGMTNNLQGSNTYALENTMIFNSQPANPGTTA
MV50  481 VNRASVSAFATTNRMELEGASYQVPPQPNGMTNNLQGSNTYALENTMIFNSQPANPGTTA
MV53  481 VNRASVSAFATTNRMELEGASYQVPPQPNGMTNNLQGSNTYALENTMIFNSQPANPGTTA

AAV5  541 TYLEGNMLITSESETQPVNRVAYNVGGQMATNNQSSTTAPATGTYNLQEIVPGSVWMERD
MV1   541 TYLEGNMLITSESETQPVNRVAYNVGGQMATNNQSSTTAPATGTYNLQEIVPGSVWMERD
MV18  541 TYLEGNMLITSESETQPVNRVAYNVGGQMATNNQSSTTAPATGTYNLQEIVPGSVWMERD
MV20  541 TYLEGNMLITSESETQPVNRVAYNVGGQMATNNQSSTTTPATGTYNLQEIVPGSVWMERD
MV50  541 TYLEGNMLITSESETQPVNRVAYNVGGQMATNNQSSTTAPATGTYNLQEIVPGSVWMERD
MV53  541 TYLEGNMLITSESETQPVNRVAYNVGGQMATNNQSSTTAPATGTYNLQEIVPGSVWMERD

AAV5  601 VYLQGPIWAKIPETGAHFHPSPAMGGFGLKHPPPMMLIKNTPVPGNITSFSDVPVSSFIT
MV1   601 VYLQGPIWAKIPETGAHFHPSPAMGGFGLKHPPPMMLIKNTPVPGNITSFSDVPVSSFIT
MV18  601 VYLQGPIWAKIPETGAHFHPSPAMGGFGLKHPPPMMLIKNTPVPGNITSFSDVPVSSFIT
MV20  601 VYLQGPIWAKIPETGAHFHPSPAMGGFGLKHPPPMMLIKNTPVPGNITSFSDVPVSSFIT
MV50  601 VYLQGPIWAKIPETGAHFHPSPAMGGFGLKHPPPMMLIKNTPVPGNITSFSDVPVSSFIT
MV53  601 VYLQGPIWAKIPETGAHFHPSPAMGGFGLKHPPPMMLIKNTPVPGNITSFSDVPVSSFIT

AAV5  661 QYSTGQVTVEMEWELKKENSKRWNPEIQYTNNYNDPQFVDFAPDSTGEYRTTRPIGTRYL
MV1   661 QYSTGQVTVEMEWELKKENSKRWNPEIQYTNNYNDPQFVDFAPDSTGEYRTTRPIGTRYL
MV18  661 QYSTGQVTVEMEWELKKENSKRWNPEIQYTNNYNDPQFVDFAPDSTGEYRTTRPIGTRYL
MV20  661 QYSTGQVTVEMEWELKKENSKRWNPEIQYTNNYNDPQFVDFAPDSTGEYRTTRPIGTRYL
MV50  661 QYSTGQVTVEMEWELKKENSKRWNPEIQYTNNYNDPQFVDFAPDGTGEYRTTRPIGTRYL
MV53  661 QYSTGQVTVEMEWELKKENSKRWNPEIQYTNNYNDPQFVDFAPDSTGEYRTTRPIGTRYL

AAV5  721 TRPL
MV1   721 TRPL
MV18  721 TRPL
MV20  721 TRPL
MV50  721 TRPL
MV53  721 TRPL
```

Figure S2: Amino Acid Sequence Alignment of AAV5 and MV Mutants

Alignment of the amino acid sequences for each of the MV mutants and AAV5. The mutations that differ from AAV5 are highlighted in black with white font.

```
AAV1    MAADGYLPDWLEDNLSEGIREWWDLKPGAPKPKANQQKQDDGRGLVLPGYKYLGPFNGLD
AAV6    MAADGYLPDWLEDNLSEGIREWWDLKPGAPKPKANQQKQDDGRGLVLPGYKYLGPFNGLD
AAV2    MAADGYLPDWLEDTLSEGIRQWWKLKPGPPPPKPAERHKDDSRGLVLPGYKYLGPFNGLD
AAV3    MAADGYLPDWLEDNLSEGIREWWALKPGVPQPKANQQHQDNRRGLVLPGYKYLGPGNGLD
AAV7    MAADGYLPDWLEDNLSEGIREWWDLKPGAPKPKANQQKQDNGRGLVLPGYKYLGPFNGLD
AAV8    MAADGYLPDWLEDNLSEGIREWWALKPGAPKPKANQQKQDDGRGLVLPGYKYLGPFNGLD
AAV9    MAADGYLPDWLEDNLSEGIREWWALKPGAPQPKANQQHQDNARGLVLPGYKYLGPGNGLD
AAV4    -MTDGYLPDWLEDNLSEGVREWWALQPGAPKPKANQQHQDNARGLVLPGYKYLGPGNGLD
MV      MSFVDHPPDWLEEVG-EGLREFLGLEAGPPKPKPNQQHQDQARGLVLPGYNYLGPGNGLD
            .: *****:   **:*::  *:.* * **. ::::*: ********:**** ****

AAV1    KGEPVNAADAAALEHDKAYDQQLKAGDNPYLRYNHADAEFQERLQEDTSFGGNLGRAVFQ
AAV6    KGEPVNAADAAALEHDKAYDQQLKAGDNPYLRYNHADAEFQERLQEDTSFGGNLGRAVFQ
AAV2    KGEPVNEADAAALEHDKAYDRQLDSGDNPYLKYNHADAEFQERLKEDTSFGGNLGRAVFQ
AAV3    KGEPVNEADAAALEHDKAYDQQLKAGDNPYLKYNHADAEFQERLQEDTSFGGNLGRAVFQ
AAV7    KGEPVNAADAAALEHDKAYDQQLKAGDNPYLRYNHADAEFQERLQEDTSFGGNLGRAVFQ
AAV8    KGEPVNAADAAALEHDKAYDQQLQAGDNPYLRYNHADAEFQERLQEDTSFGGNLGRAVFQ
AAV9    KGEPVNAADAAALEHDKAYDQQLKAGDNPYLKYNHADAEFQERLKEDTSFGGNLGRAVFQ
AAV4    KGEPVNAADAAALEHDKAYDQQLKAGDNPYLKYNHADAEFQQRLQGDTSFGGNLGRAVFQ
MV      RGEPVNRADEVAREHDISYNEQLEAGDNPYLKYNHADAEFQEKLADDTSFGGNLGKAVFQ
        :***** ** .* *** :*:.**.:******:*********::*  *********:****

AAV1    AKKRVLEPLGLVEEGAKTAPGKKRPVEQSPQE-PDSSSGIGKTGQQPAKKRLNFGQTGDS
AAV6    AKKRVLEPFGLVEEGAKTAPGKKRPVEQSPQE-PDSSSGIGKTGQQPAKKRLNFGQTGDS
AAV2    AKKRVLEPLGLVEEPVKTAPGKKRPVEHSPVE-PDSSSGTGKAGQQPARKRLNFGQTGDA
AAV3    AKKRILEPLGLVEEAAKTAPGKKRPVDQSPQE-PDSSSGVGKSGKQPARKRLNFGQTGDS
AAV7    AKKRVLEPLGLVEEGAKTAPAKKRPVEPSPQRSPDSSTGIGKKGQQPARKRLNFGQTGDS
AAV8    AKKRVLEPLGLVEEGAKTAPGKKRPVEPSPQRSPDSSTGIGKKGQQPARKRLNFGQTGDS
AAV9    AKKRLLEPLGLVEEAAKTAPGKKRPVEQSPQE-PDSSAGIGKSGAQPAKKRLNFGQTGDT
AAV4    AKKRVLEPLGLVEQAGETAPGKKRPLIESPQQ-PDSSTGIGKKGKQPAKKKLVFEDETGA
MV      AKKRVLEPFGLVEEGAKTAPTGKRIDDHFPKR----------KKARTEEDSKPSTSSDAE
        ****:***:****:  :***  **     * .             :. ..     .

AAV1    ESVPDPQPLGEPPATPAAVGPTTMASGGGAPMADNNEGADGVGNASGNWHCDSTWLGDRV
AAV6    ESVPDPQPLGEPPATPAAVGPTTMASGGGAPMADNNEGADGVGNASGNWHCDSTWLGDRV
AAV2    DSVPDPQPLGQPPAAPSGLGTNTMATGSGAPMADNNEGADGVGNSSGNWHCDSTWMGDRV
AAV3    ESVPDPQPLGEPPAAPTSLGSNTMASGGGAPMADNNEGADGVGNSSGNWHCDSQWLGDRV
AAV7    ESVPDPQPLGEPPAAPSSVGSGTVAAGGGAPMADNNEGADGVGNASGNWHCDSTWLGDRV
AAV8    ESVPDPQPLGEPPAAPSGVGPNTMAAGGGAPMADNNEGADGVGSSSGNWHCDSTWLGDRV
AAV9    ESVPDPQPIGEPPAAPSGVGSLTMASGGGAPVADNNEGADGVGSSSGNWHCDSQWLGDRV
AAV4    GDGPP-----EGSTSGAMSDDSEMRAAAGGAAVEGGQGADGVGNASGDWHCDSTWSEGHV
MV      AGPSGSQQLRIPAQPASSLGADTMSAGGGGPLGDNNQGADGVGNASGDWHCDSTWMGDRV
         . .        . . :  .   : :..*..  :..:******.:**:***** *   .:*

AAV1    ITTSTRTWALPTYNNHLYK---QIS-SASTGASNDNHYFGYSTPWGYFDFNRFHCHFSPR
AAV6    ITTSTRTWALPTYNNHLYK---QIS-SASTGASNDNHYFGYSTPWGYFDFNRFHCHFSPR
AAV2    ITTSTRTWALPTYNNHLYK---QIS-SQSG-ASNDNHYFGYSTPWGYFDFNRFHCHFSPR
AAV3    ITTSTRTWALPTYNNHLYK---QIS-SQSG-ASNDNHYFGYSTPWGYFDFNRFHCHFSPR
AAV7    ITTSTRTWALPTYNNHLYK---QISSETAG-STNDNTYFGYSTPWGYFDFNRFHCHFSPR
AAV8    ITTSTRTWALPTYNNHLYK---QISNGTSGGATNDNTYFGYSTPWGYFDFNRFHCHFSPR
AAV9    ITTSTRTWALPTYNNHLYK---QISNSTSGGSSNDNAYFGYSTPWGYFDFNRFHCHFSPR
AAV4    TTTSTRTWVLPTYNNHLYKRLG--------ESLQSNTYNGFSTPWGYFDFNRFHCHFSPR
MV      VTKSTRTWVLPSYNNHQYR----EIKSGSVDRSNANAYFGYSTPWGYFDFNRFHSHWSPR
         *.*****.**:**** *:              : * * *:*************.*:***

AAV1    DWQRLINNNWGFRPKRLNFKLFNIQVKEVTTNDGVTTIANNLTSTVQVFSDSEYQLPYVL
AAV6    DWQRLINNNWGFRPKRLNFKLFNIQVKEVTTNDGVTTIANNLTSTVQVFSDSEYQLPYVL
AAV2    DWQRLINNNWGFRPKRLNFKLFNIQVKEVTQNDGTTTIANNLTSTVQVFTDSEYQLPYVL
AAV3    DWQRLINNNWGFRPKKLSFKLFNIQVKEVTQNDGTTTIANNLTSTVQVFTDSEYQLPYVL
AAV7    DWQRLINNNWGFRPKKLRFKLFNIQVKEVTTNDGVTTIANNLTSTIQVFSDSEYQLPYVL
AAV8    DWQRLINNNWGFRPKRLSFKLFNIQVKEVTQNEGTKTIANNLTSTIQVFTDSEYQLPYVL
AAV9    DWQRLINNNWGFRPKRLNFKLFNIQVKEVTDNNGVKTIANNLTSTVQVFTDSDYQLPYVL
AAV4    DWQRLINNNWGMRPKAMRVKIFNIQVKEVTTSNGETTVANNLTSTVQIFADSSYELPYVM
```

```
MV              DWQRLINNYWGFRPRSLRVKIFNIQVKEVTVQDSTTTIANNLTSTVQVFTDDDYQLPYVV
                ******** **:**: : .*:********* .:. .*:*******:*:*:*..*:****:

AAV1            GSAHQGCLPPFPADVFMIPQYGYLTLNNG---SQAVGRSSFYCLEYFPSQMLRTGNNFTF
AAV6            GSAHQGCLPPFPADVFMIPQYGYLTLNNG---SQAVGRSSFYCLEYFPSQMLRTGNNFTF
AAV2            GSAHQGCLPPFPADVFMVPQYGYLTLNNG---SQAVGRSSFYCLEYFPSQMLRTGNNFTF
AAV3            GSAHQGCLPPFPADVFMVPQYGYLTLNNG---SQAVGRSSFYCLEYFPSQMLRTGNNFQF
AAV7            GSAHQGCLPPFPADVFMIPQYGYLTLNNG---SQSVGRSSFYCLEYFPSQMLRTGNNFEF
AAV8            GSAHQGCLPPFPADVFMIPQYGYLTLNNG---SQAVGRSSFYCLEYFPSQMLRTGNNFQF
AAV9            GSAHEGCLPPFPADVFMIPQYGYLTLNDG---SQAVGRSSFYCLEYFPSQMLRTGNNFQF
AAV4            DAGQEGSLPPFPNDVFMVPQYGYCGLVTGNTSQQQTDRNAFYCLEYFPSQMLRTGNNFEI
MV              GNGTEGCLPAFPPQVFTLPQYGYATLNRDN-TENPTERSSFFCLEYFPSKMLRTGNNFEF
                . . :*.**.** :** :*****  *  .   .: . *.:*:*******:******** :

AAV1            SYTFEEVPFHSSYAHSQSLDRLMNPLIDQYLYYLNRTQ-NQSGSAQNKDLLFSRGSPAGM
AAV6            SYTFEDVPFHSSYAHSQSLDRLMNPLIDQYLYYLNRTQ-NQSGSAQNKDLLFSRGSPAGM
AAV2            SYTFEDVPFHSSYAHSQSLDRLMNPLIDQYLYYLSRTN-TPSGTTTQSRLQFSQAGASDI
AAV3            SYTFEDVPFHSSYAHSQSLDRLMNPLIDQYLYYLNRTQGTTSGTTNQSRLLFSQAGPQSM
AAV7            SYSFEDVPFHSSYAHSQSLDRLMNPLIDQYLYYLARTQSNPGGTAGNRELQFYQGGPSTM
AAV8            TYTFEDVPFHSSYAHSQSLDRLMNPLIDQYLYYLSRTQTTGG-TANTQTLGFSQGGPNTM
AAV9            SYEFENVPFHSSYAHSQSLDRLMNPLIDQYLYYLSKTINGSG--QNQQTLKFSVAGPSNM
AAV4            TYSFEKVPFHSMYAHSQSLDRLMNPLIDQYLWGLQSTTTGTTLNAGTATTNFTKLRPTNF
MV              TYNFEEVPFHSSLAPSQNLFKLANPLVDQYLYRFVSTN-------NTGGVQFNKNLAGRY
                :* **.*****  * **.* :* ***:****: :  *              *    .

AAV1            SVQPKNWLPGPCYRQQRVSKTKTD-----NNNSNFTWTGASKYNLNGRESIINPGTAMAS
AAV6            SVQPKNWLPGPCYRQQRVSKTKTD-----NNNSNFTWTGASKYNLNGRESIINPGTAMAS
AAV2            RDQSRNWLPGPCYRQQRVSKTSAD-----NNNSEYSWTGATKYHLNGRDSLVN--PAMAS
AAV3            SLQARNWLPGPCYRQQRLSKTAND-----NNNSNFPWTAASKYHLNGRDSLVNPGPAMAS
AAV7            AEQAKNWLPGPCFRQQRVSKTLDQ-----NNNSNFAWTGATKYHLNGRNSLVNPGVAMAT
AAV8            ANQAKNWLPGPCYRQQRVSTTTGQ-----NNNSNFAWTAGTKYHLNGRNSLANPGIAMAT
AAV9            AVQGRNYIPGPSYRQQRVSTTVTQ-----NNNSEFAWPGASSWALNGRNSLMNPGPAMAS
AAV4            SNFKKNWLPGPSIKQQGFSKTANQNYKIPATGSDSLIKYETHSTLDGRWSALTPGPPMAT
MV              ANTYKNWFPGPMGRTQGWNLGSGVN-----RASVSAFATTNRMELEGASYQVPPQPNGMT
                    :*::***  : *  .             *       .   *:*            :

AAV1            HKDDEDKFFPMSGVMIFGKESAGA---SNTALDNVMITDEEEIKATNPVATERFGTVAVN
AAV6            HKDDKDKFFPMSGVMIFGKESAGA---SNTALDNVMITDEEEIKATNPVATERFGTVAVN
AAV2            HKDDEEKFFPQSGVLIFGKQGSEK---TNVNIEKVMITDEEEIGTTNPVATEQYGSVSTN
AAV3            HKDDEEKFFPMHGNLIFGKEGTTA---SNAELDNVMITDEEEIRTTNPVATEQYGTVANN
AAV7            HKDDEDRFFPSSGVLIFG-KTGAT---NKTTLENVLMTNEEEIRPTNPVATEEYGIVSSN
AAV8            HKDDEERFFPSNGILIFGKQNAAR---DNADYSDVMLTSEEEIKTTNPVATEEYGIVADN
AAV9            HKEGEDRFFPLSGSLIFGKQGTGR---DNVDADKVMITNEEEIKTTNPVATESYGQVATN
AAV4            AGPADSKFS-NSQLIFAGPKQNGN---TATVPGTLIFTSEEELAATNATDTDMWGNLPGG
MV              NNLQGSNTYALENTMIFNSQPANPGTTATYLEGNMLITSESETQPVNRVAYNVGGQMATN
                     ..       :: . :              :::*.*.*  ..* .  :  * :. .

AAV1            FQSSSTDPATGDVHAMGALPGMVWQDRDVYLQGPIWAKIPHTDGHFHPSPLMGGFGLKNP
AAV6            LQSSSTDPATGDVHVMGALPGMVWQDRDVYLQGPIWAKIPHTDGHFHPSPLMGGFGLKHP
AAV2            LQRGNRQAATADVNTQGVLPGMVWQDRDVYLQGPIWAKIPHTDGHFHPSPLMGGFGLKHP
AAV3            LQSSNTAPTTRTVNDQGALPGMVWQDRDVYLQGPIWAKIPHTDGHFHPSPLMGGFGLKHP
AAV7            LQAANTAAQTQVVNNQGALPGMVWQNRDVYLQGPIWAKIPHTDGNFHPSPLMGGFGLKHP
AAV8            LQQQNTAPQIGTVNSQGALPGMVWQNRDVYLQGPIWAKIPHTDGNFHPSPLMGGFGLKHP
AAV9            HQSAQAQAQTGWVQNQGILPGMVWQDRDVYLQGPIWAKIPHTDGNFHPSPLMGGFGMKHP
AAV4            DQSNSNLPTVDRLTALGAVPGMVWQNRDIYYQGPIWAKIPHTDGHFHPSPLIGGFGLKHP
MV              NQSSTTTPATGTYNLQEIVPGSVWMERDVYLQGPIWAKIPETGAHFHPSPAMGGFGLKHP
                 *     .          :** ** :**:* *********.*..:***** :****:*:*

AAV1            PPQILIKNTPVPANPPAEFSATKFASFITQYSTGQVSVEIEWELQKENSKRWNPEVQYTS
AAV6            PPQILIKNTPVPANPPAEFSATKFASFITQYSTGQVSVEIEWELQKENSKRWNPEVQYTS
AAV2            PPQILIKNTPVPANPSTTFSAAKFASFITQYSTGQVSVEIEWELQKENSKRWNPEIQYTS
AAV3            PPQIMIKNTPVPANPPTTFSPAKFASFITQYSTGQVSVEIEWELQKENSKRWNPEIQYTS
AAV7            PPQILIKNTPVPANPPEVFTPAKFASFITQYSTGQVSVEIEWELQKENSKRWNPEIQYTS
```

```
AAV8            PPQILIKNTPVPADPPTTFNQSKLNSFITQYSTGQVSVEIEWELQKENSKRWNPEIQYTS
AAV9            PPQILIKNTPVPADPPTAFNKDKLNSFITQYSTGQVSVEIEWELQKENSKRWNPEIQYTS
AAV4            PPQIFIKNTPVPANPATTFSSTPVNSFITQYSTGQVSVQIDWEIQKERSKRWNPEVQFTS
MV              PPMMLIKNTPVPGN-ITSFSDVPVSSFITQYSTGQVTVEMEWELKKENSKRWNPEIQYTN
                ** ::*******.:    *.   . ***********:*:::**::**.*******:*:*.

AAV1            NYAKSANVDFTVDNNGLYTEPRPIGTRYLTRPL
AAV6            NYAKSANVDFTVDNNGLYTEPRPIGTRYLTRPL
AAV2            NYNKSVNRGLTVDTNGVYSEPRPIGTRYLTRNL
AAV3            NYNKSVNVDFTVDTNGVYSEPRPIGTRYLTRNL
AAV7            NFEKQTGVDFAVDSQGVYSEPRPIGTRYLTRNL
AAV8            NYYKSTSVDFAVNTEGVYSEPRPIGTRYLTRNL
AAV9            NYYKSNNVEFAVNTEGVYSEPRPIGTRYLTRNL
AAV4            NYGQQNSLLWAPDAAGKYTEPRAIGTRYLTHHL
MV              NYNDPQFVDFAPDGTGEYRSTRPIGTRYLTRPL
                *: .      : :  * * ..*.*******: *
```

Figure S3: Alignment of All Mutations found in MV Mutants with Other AAV Serotypes

Alignment of AAV serotypes 1 through 9 against MV which contains all of the mutations found in the MV mutants pooled into one sequence. Mutations are highlighted in red.

A

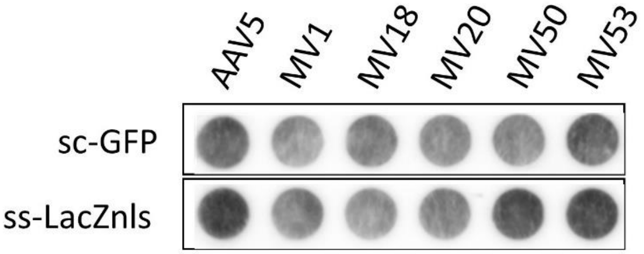

B

AAV5 and MV Mutant Viral Titer

Virus	sc-GFP (vg/ml)	ss-LacZ (vg/ml)
AAV5	2.10E+12	3.78E+12
MV1	1.35E+12	1.46E+12
MV18	1.50E+12	1.96E+12
MV20	1.40E+12	2.36E+12
MV50	1.45E+12	3.54E+12
MV53	2.30E+12	3.76E+12

Figure S4: Production Yield of AAV5 and MV Mutants

(A) DNA dotblot for the yield of each mutant and AAV5 for two versions of the virus, sc-GFP and ss-LacZnls. (B) Production titers for each capsid and each transgene version.

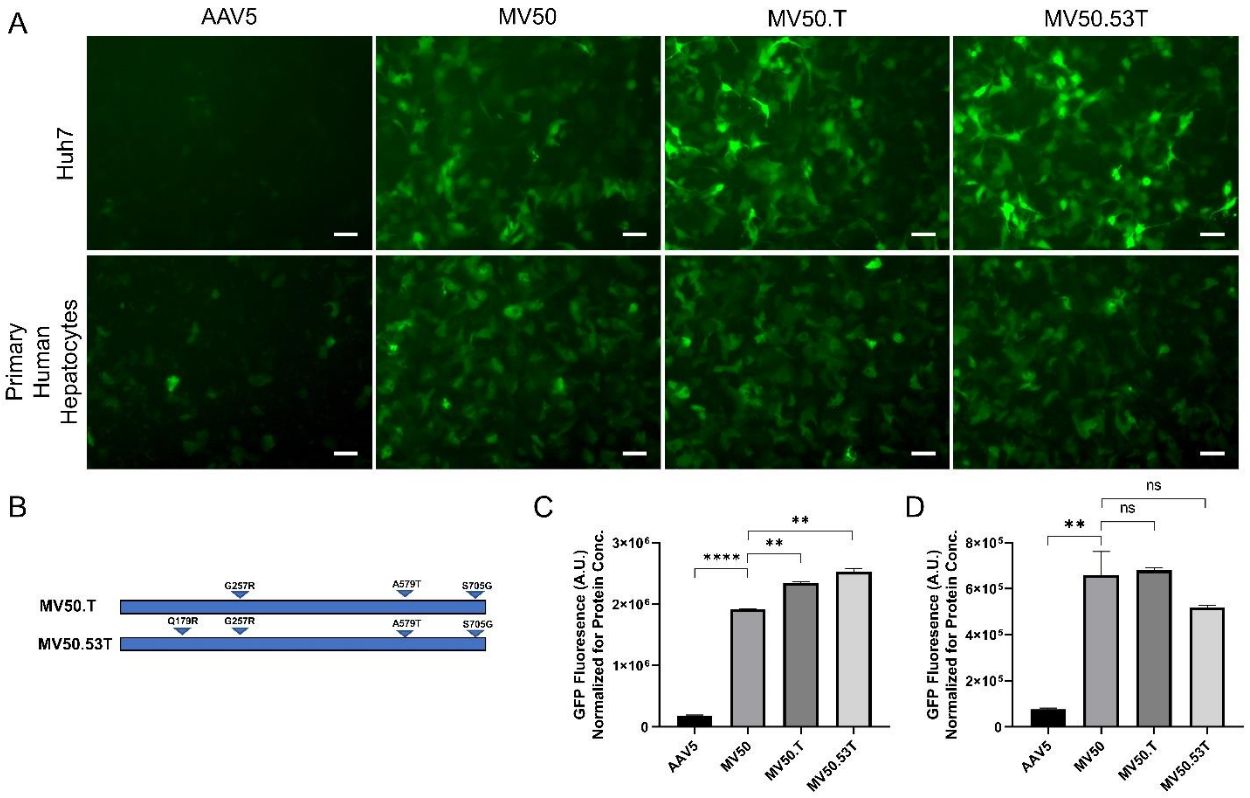

Figure S5. Combination of Mutations from multiple AAV5 Variants

(A) Fluorescent imaging of Huh7 cells and primary human hepatocytes(TRL HUM4020) transduced with self-complementary GFP vectors with AAV5, MV50, MV50.T, and MV50.53T. Huh7 cells were transduced at a MOI of 1e5 vg/cell and human hepatocytes were transduced at a MOI of 5e5vg/cell. Images were taken 96 hours after infection. Expression of GFP (green) indicates transduced cells. 10x magnification. Scale Bar: 100um. (B) Depiction of the combined mutations that are in MV50.T and MV50.53T. Location of amino acid mutations in the AAV5 VP1 amino acid sequence are indicated by triangles. The exact mutation is indicated above the triangle. (C) Comparison of normalized GFP expression in Huh7 cells between AAV5, MV50, MV50.T, and MV50.53T. Data are shown as mean values ± SD. **p<0.01; ****p<0.0001. (D) Comparison of normalized GFP expression in primary human hepatocytes between AAV5, MV50, MV50.T, and MV50.53T. Data are shown as mean values ± SD. **p<0.01.

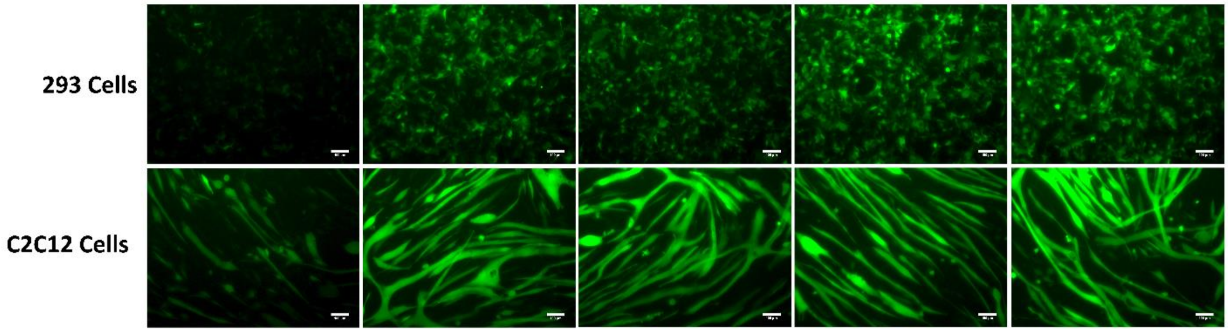

Figure S6. Transduction Efficiency of MV Mutants Compared to AAV5 in 293 and Differentiated C2C12 Cells

(A) Fluorescent imaging of 293 cells and differentiated C2C12 cells transduced with self-complementary GFP vectors packaged by AAV5, MV1, MV18, MV50, and MV53. Both cell lines were transduced at a MOI of 4e5 vg/cell. Images were taken 96 hours after infection. Expression of GFP (green) indicates transduced cells. 10x magnification. Scale Bar: 100um.

Table S1: Replacement of AAV5 Loop1/VRI Amino Acid Sequence with Other AAV Serotype Loop1

AAV Serotype	Loop1 Sequence	Packages DNA?	Infectious on 293?	Infectious in Mice?
1	ISSASTGASNDNHY	No	No	No
2	ISSQSGASNDNHY	No	No	No
4	LGESLQSNTYN	No	No	No
7	ISSETAGSTNDNTY	Yes	No	No
8	ISNGTSGGATNDNTY	No	No	No
9	ISNGTSGGSTNDNTY	No	No	No
Avian	IQGPGGTDPNNKFF	Yes	No	No

Replacement of AAV5 Loop1/VRI amino acid sequence with other AAV serotypes Loop1/VRI sequence. The sequence IKSGSVDGSNANAY was replaced with the sequence indicated in the table using PCR-based cloning. Modified AAV5 capsids were then used to package DNA and tested for their infectivity in 293 cells and mice.

Supplemental Methods

Transduction of 293 Cells by AAV Vectors

Variant AAV5 vectors were used to packaged sc-GFP reporter genes for comparison to wt-AAV5. 293 cells in 12 well plates were infected with AAV5 and mutant self-complementary(sc)-GFP encoding viruses at a MOI of 400,000 viral genomes(vg) per cell and co-infected with 5000 vg/cell of wt-ad5. Ninety-six hours after infection, cells were taken for fluorescent imaging.

Transduction of Differentiated C2C12 Cells by AAV Vectors

Variant AAV5 vectors were used to packaged sc-GFP reporter genes for comparison to wt-AAV5. C2C12 myoblasts were seeded in 12 well plates were cultured with DMEM supplemented with 10% FBS and 1% penicillin/streptomycin (p/s). Upon reaching 70% confluency, culture medium was changed to differentiated medium (DMEM with 2% horse serum and 1% p/s). C2C12 cells were differentiated for five days prior to infection with AAV5 and mutant self-complementary(sc)-GFP encoding viruses at a MOI of 400,000 viral genomes(vg) per cell and co-infected with 5000 vg/cell of wt-ad5. Ninety-six hours after infection, cells were taken for fluorescent imaging. Differentiation medium was changed every two days.
