# Supplementary material for: Directed Evolution of AAV Serotype 5 for Increased Hepatocyte Transduction and Retained Low Humoral Seroreactivity
Source: Mol Ther Methods Clin Dev. 2020 Oct 20;20:122–32. doi: 10.1016/j.omtm.2020.10.010 (PMC7773954; doi:10.1016/j.omtm.2020.10.010)
Supplement: Document S2. Article plus Supplemental Information [file mmc2.pdf]

Please cite this article in press as: Qian et al., Directed Evolution of AAV Serotype 5 for Increased Hepatocyte Transduction and Retained Low Humoral Seroreactivity, Molecular Therapy: Methods & Clinical Development (2020), https://doi.org/10.1016/j.omtm.2020.10.010

Molecular Therapy
Methods & Clinical Development
Original Article

Directed Evolution of AAV Serotype 5 for Increased Hepatocyte Transduction and Retained Low Humoral Seroreactivity

Q1 Randolph Qian,[1] Bin Xiao,[1] Juan Li,[1] and Xiao Xiao[1]

[1]Division of Pharmacoengineering and Molecular Pharmaceutics, Eshelman School of Pharmacy, University of North Carolina, Chapel Hill, NC 27517, USA

Most recombinant adeno-associated virus (AAV) capsids utilized in liver gene therapy have significant levels of pre-existing neutralizing antibodies in the human population. These neutralizing factors limit the patient pools eligible for receiving AAV-mediated therapies. AAV serotype 5 (AAV5) does not face the same barrier of humoral immunity as most AAV serotypes due to its low seroprevalence. However, AAV5 can only facilitate a low level of transgene expression in the liver, constraining its application to a small number of liver diseases. To improve the liver transduction of AAV5 while retaining its low seroprevalence, we constructed a library of AAV5 mutants via random mutagenesis and screened in Huh7 cells. Two molecularly evolved AAV5 variants, MV50 and MV53, demonstrated significantly increased transduction efficiency in Huh7 cells (∼12×) and primary human hepatocytes (∼10×). All variants had retained low seroreactivity toward pooled human immunoglobulin G (IgG) when compared to AAV5, which was significantly less seroreactive than AAV9. Functional characterization of the mutants also revealed insights into the functions of various domains, especially the VR-I, in the AAV5 capsid. The result is AAV5 variant capsids with much enhanced human hepatocyte transduction, potentially useful for liver-directed gene therapy.

Q7 Q8 Q2 INTRODUCTION

Q4 Q5 Q6 Q3 Development of adeno-associated virus (AAV) gene therapies for liver diseases has made substantial progress in the past decade, culminating in landmark clinical trials for diseases such as hemophilia A and B.[1–3] However, successful clinical trials have mainly been limited to liver diseases that only require a small fraction of normal protein expression for therapeutic effect, which is readily achievable by administering moderately high doses of hepatotropic recombinant AAV (rAAV) capsids.[4–6] Treatment of liver diseases requiring more extensive transduction of human hepatocytes remains a challenge as many of the original hepatotropic rAAV capsids cannot achieve the high levels of gene expression needed for therapeutic efficacy.[4]

As such, many studies have focused on developing engineered rAAV capsids through directed evolution or rational design that exceed the transduction properties of traditional AAV serotypes.[7–9] Through these methods, AAV can be modified to transduce new cell types or enhance existing tropism. This has resulted in novel AAV capsids with substantially improved human liver transduction capabilities with the potential of treating a wider range of liver diseases.[7,8] However, engineered capsids often face the same barrier of humoral immunity as natural AAV serotypes due to similarities in structure and sequence.[10] Most naturally isolated AAV serotypes utilized for hepatic delivery have significant levels of pre-existing neutralizing antibodies in the human population, limiting the patient pools eligible for receiving rAAV based therapies.[11–13] Pre-existing neutralizing antibodies against AAV can often cross neutralize many different AAV serotypes through conserved sequences on the capsid surface.[14] Thus, patients with prior exposure to natural AAV capsids will likely have antibodies that also neutralize engineered AAV capsids due to cross-neutralization. Low titers of anti-AAV antibodies are sufficient to neutralize systemic rAAV and prevent transgene expression, leading pre-existing antibodies to be a major barrier for both natural and engineered AAV capsids.[15,16]

Interestingly, most evolved capsids contain little to no traces of AAV5 sequences, likely a result of AAV5 having the most divergent sequence of all AAV serotypes.[17] AAV5 is particularly divergent in regions that constitute the exterior surface of the capsid, leading to significant differences in receptor engagement, which may explain why AAV5 demonstrates significantly less transduction in human hepatocytes when compared to other serotypes such as AAV3b and AAV8.[8,9,17] As such, AAV5 can only facilitate low levels of transgene expression in the human liver, limiting its application to diseases such as hemophilia A and B. However, AAV5 has been consistently characterized as the serotype with the lowest seroprevalence of pre-existing neutralizing factors, giving it a major advantage versus other AAV serotypes in overcoming the barrier of pre-existing antibodies.[11,18] Studies utilizing serum from healthy adult donors in Europe and America concluded that as low as ∼3% and ∼13% of their respective study population tested positive for neutralizing factors against

Received 26 July 2020; accepted 13 October 2020;
https://doi.org/10.1016/j.omtm.2020.10.010.

Correspondence: Xiao Xiao, Division of Pharmacoengineering and Molecular Pharmaceutics, Eshelman School of Pharmacy, University of North Carolina, Chapel Hill, NC 27517, USA.
E-mail: xxiao@email.unc.edu

This is an open access article under the CC BY-NC-ND license (http://creativecommons.org/licenses/by-nc-nd/4.0/).

Please cite this article in press as: Qian et al., Directed Evolution of AAV Serotype 5 for Increased Hepatocyte Transduction and Retained Low Humoral Seroreactivity, Molecular Therapy: Methods & Clinical Development (2020), https://doi.org/10.1016/j.omtm.2020.10.010

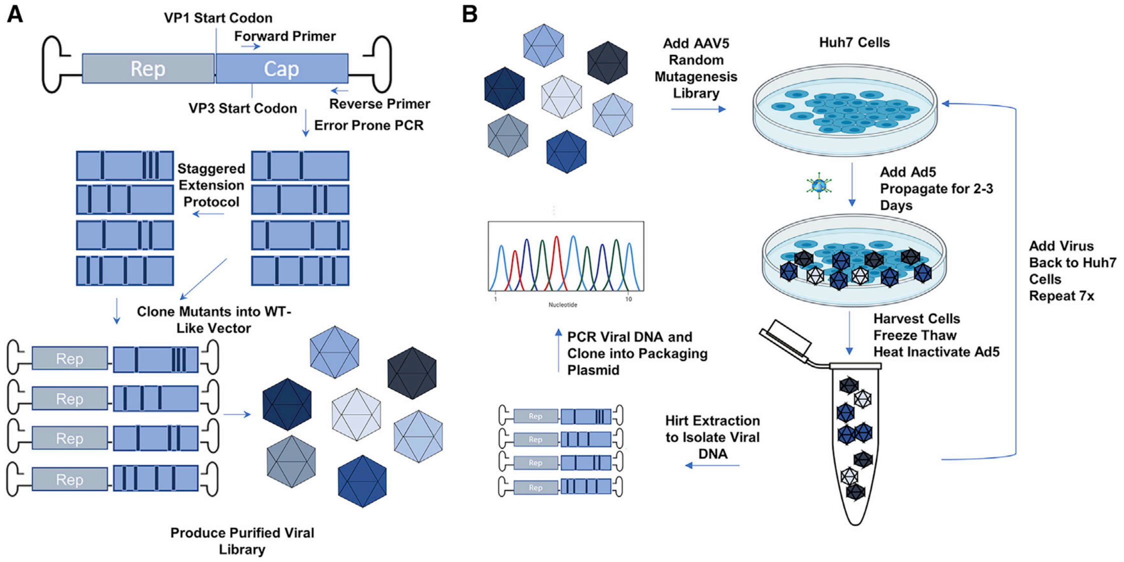

Figure 1. Directed Evolution of Adeno-Associated Virus Serotype 5 Capsid by Random Mutagenesis and Sequential Screening in Huh7 Cells

(A) Diagram illustrating the mutant AAV5 library creation process. WT-AAV5 capsids genes were PCR-amplified using an error prone polymerase and subsequently shuffled using the staggered extension protocol (stEP). The error prone and stEP amplicon libraries were pooled and cloned into a WT-like vector plasmid that could produce replicant competent AAV vectors. The plasmid library was then amplified and used to produce a purified AAV5 library using the cesium chloride density ultracentrifugation method. (B) Diagram depicting the screening process. Purified mutated AAV5 library was added to Huh7 cells, which were coinfected with WT-adenovirus type 5 to allow for replicative screening. Cells were harvested along with the supernatant and lysed using the freeze-thaw method. The lysate was centrifuged and the supernatant with the mutant AAV5 capsids was taken for the next round of screening. The screening process was repeated a total of 7 times. In the very last round of selection, the mutant AAV5 capsid DNA were extracted and amplified. After cloning into a packaging plasmid containing AAV2 rep, the plasmids were taken for amplification and screening.

AAV5, which was significantly lower compared to other serotypes such as AAV2, AAV6, and AAV8.[11,12] Additionally, clinical trials utilizing AAV5 for hemophilia A have noted that patients with pre-existing anti-AAV5 antibodies had sustained levels of FVIII expression comparable to patients without neutralizing factors which indicates that AAV5 could be used even in the presence of neutralizing factors in serum.[2,19] Thus, evolved AAV5 vectors that have enhanced transduction capabilities could have tremendous advantages in evading neutralizing antibodies and improving liver gene therapy.

In this study, we engineered diverse libraries of AAV5 mutants using error prone pcr and the staggered extension protocol using previously established methods.[20–22] A replicating AAV5 library was successfully screened in Huh7 cells, a human liver carcinoma cell line, resulting in mutant AAV5 capsids that exhibited increased transduction in human hepatocytes and retained similar seroreactivity compared to AAV5. Functional characterization of the mutations in selected AAV5 variants provided insights into various domains in the AAV5 capsid. The result is AAV5 mutant capsids that have enhanced transduction in human hepatocytes and favorable seroreactivities.

RESULTS

Generation and Screening of a Diverse AAV5 Capsid Library in Huh7 Cell Line

To evolve AAV5 for enhanced liver gene therapy, we constructed a diverse library of AAV5 capsid genes by applying a similar method previously described by Maheshri et al.[22] in which error prone PCR (EPPCR) and staggered extension protocol (stEP) generated random point mutations in the AAV5 VP3 gene. Diversely mutated AAV5 capsid genes were cloned into an infectious vector containing AAV2 ITRs and a chimeric AAV2 and AAV5 rep gene. An infectious wild-type (WT)-like AAV5 library was produced using the triple plasmid transfection method followed by purification via cesium chloride density ultracentrifugation (Figure 1A). The AAV5 library was screened on Huh7 cells, a human hepatocyte derived carcinoma cell line, for seven rounds of selection. Viral DNA was extracted from a portion of the cells at rounds 5 and 7 via a modified Hirt Extraction and the isolated VP3 genes were amplified, cloned into a packaging plasmid, and sequenced (Figure 1B).

Sequencing of AAV5 capsids selected from the 5th round of selection revealed that approximately half (31 out of 65) of the sequenced mutants contained an glycine to arginine (G257R), or "Loop1R" mutation in the variable region I (VR I) of the AAV5 VP1 protein (Figure 2A). Further enrichment of the mutants carrying the Loop1R mutation was observed after the 7th round of selection where 73 out of 75 mutants carried the Loop1R mutation (Figure 2A). All unique sequences obtained from the 5th and 7th rounds of selection were used to package self-complementary GFP (sc-GFP) vector and crudely assessed for infectivity and yield. The five best variants were selected for large scale production and purification for further analysis.

Please cite this article in press as: Qian et al., Directed Evolution of AAV Serotype 5 for Increased Hepatocyte Transduction and Retained Low Humoral Seroreactivity, Molecular Therapy: Methods & Clinical Development (2020), https://doi.org/10.1016/j.omtm.2020.10.010

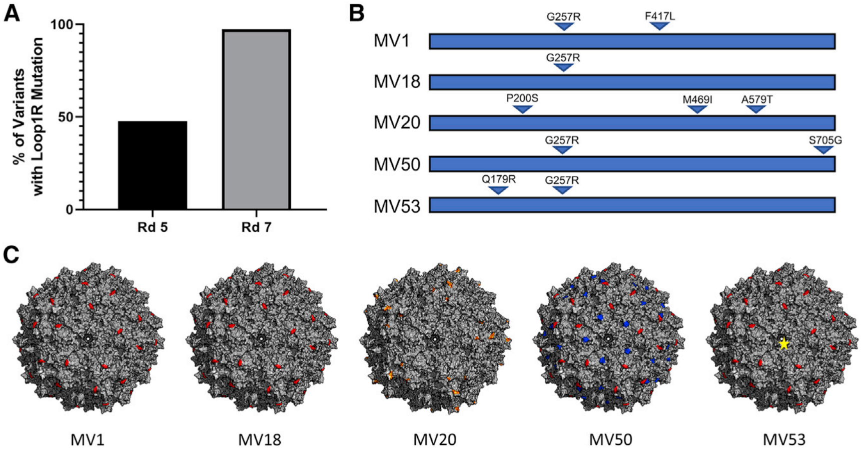

Figure 2. Selection, Sequence, and Structural Analysis of Mutant AAV5 Capsids with Increased Human Liver Transduction

(A) Percentage of sequenced AAV5 variants carrying the G257R mutation (Loop1R) from the fifth and seventh rounds of selection. (B) Five selected mutants with the best yield and transduction capabilities. Location of amino acid mutations in the AAV5 VP1 amino acid sequence are indicated by triangles. The exact mutation is indicated above the triangle. (C) Mutations for each variant were false color mapped onto the surface of the 60-mer crystal structure of AAV5. The G257R mutation is colored in red, the A579T mutation is colored in orange, and the S705G mutation is colored in blue. The F417L and M469I mutations do not show up as they are located in the interior of the MV1 and MV20 capsid. The exact structure of the domain that the Q179R resides in is unknown. The yellow star in MV53 indicates a possible location for the Q179R mutation, if the domain resides near or on the surface of the MV53 capsid. False-color mapping of the mutations onto the AAV5 capsid was exaggerated by highlighting the adjacent amino acids in addition to the mutation to allow for better visualization of the mutations on the capsid. AAV5 60-mer capsid structures were obtained by using AAV5 VP3 protein structure 3NTT and viperdb to generate a 60-mer consisting of AAV5 VP3 proteins.

Sequence and Structural Analysis of Isolated AAV5 Variants

Sequence alignment of the five variants with WT-AAV5 revealed that the selected mutations were primarily spread across the AAV5 VP3 gene, with only one variant, MV53, carrying a mutation in the VP1/VP2 common region (Figures 2B; Figure S1). Four (MV1, MV18, MV50, MV53) out of the five variants carried the Loop1R mutation, of which three mutants (MV1, MV50, MV53) were likely evolutionarily derived from a parental variant (MV18) that only carried the Loop1R mutation (Table 1; Figure S2). MV1, MV50, and MV53 sequences were not found in the sequencing data from the 5th round of selection, indicating that these variants may have arisen later in the selection process and hence derived from MV18. False-color mapping of mutations onto the crystal structure of AAV5 revealed that the Loop1R mutation is located in a surface exposed part of the VR-I region (Table 1; Figures 2B and 2C). MV1 contains a secondary mutation (F417L) that is buried in the interior of the capsid in a fairly conserved region across AAV serotypes that likely does not play a role in altering binding of AAV5 (Table 1; Figures 2B and 2C). MV50 contains a secondary mutation (S705G) near the VR IX (Loop 9) region and is present on the surface of the capsid as determined by false-color mapping (Table 1; Figures 2B and 2C). This region has also been characterized to be involved with receptor binding and is close to known antigenic sites on other serotypes.[23] MV53's secondary mutation could not be analyzed by structural analysis as the AAV5 VP1/VP2 common region and the N terminus of the AAV5 VP3 protein could not be resolved by X-ray crystallography (Figures 2B and 2C).[24] The only variant to not carry the Loop1R mutation is MV20, which instead contains three different amino acid mutations versus WT-AAV5 that are not found in any of the other variants (Table 1; Figure 2B). Only one of the three mutations (A579T) is located in a surface exposed region, VR VIII, which has been shown to be involved in binding sialic acid for AAV5 (Figure 2C).[25]

Characterizing AAV5 Variants for Increases in Human Liver Cell Transduction

We next investigated the transduction efficacy of the AAV5 variants versus WT-AAV5 in Huh7 cells. Fluorescent imaging of Huh7 cells transduced with sc-GFP versions of MV mutants and AAV5 revealed that all five mutants had significantly increased GFP expression compared to AAV5 (Figure 3A). MV50 was the best performing capsid that was around 12× better than AAV5 in transduction of Huh7 cells (Figure 3B). The second and third best variants were MV53 and MV1, which were 11× and 10× better than AAV5, respectively (Figure 3B). The non Loop1R mutant, MV20, showed a 6× increase in infectivity compared to AAV5 (Figure 3B).

Next, the five AAV5 mutants were tested for their transduction capabilities in donor primary human hepatocytes. The AAV5 mutants were all significantly better at infecting primary human hepatocytes when compared with WT-AAV5 (Figure 3A). MV50 and MV53 were the two variants with the best transduction capabilities and both infected primary human hepatocytes roughly 10-fold better compared to AAV5, which was very consistent with the fold increase in Huh7 cells (Figure 3C). The increase in infectivity of the other three mutants, MV1, MV18, and MV20 over AAV5 were again consistent with the results from Huh7 Cells (Figure 3C). The consistency of the MV mutants in Huh7 and human hepatocytes demonstrated the viability of using Huh7 cells to screen for infectivity in human hepatocytes (Figure 3D).

B-galactosidase (LacZ) reporter gene vectors were packaged by the AAV5 variants and injected intravenously into non-humanized C57BL/6 mice (1e12 vg/ mouse) to assess whether or not the variants had altered tropism in a mouse model (Figure 4A). MV1 and MV53 were the only variants that had slightly higher levels of LacZ activity in

OMTM 576

Table 1. Mutations of AAV5 Variants with Increased Liver Tropism

Variant	Mutation	Region/Probable Function
MV1	G257R	surface exposed in VR-I/Loop1 region, increases binding and internalization
	F417L	buried in interior of capsid, affects stability of capsid and perhaps uncoating
MV18	G257R	surface region in VR-I/Loop1, increases binding and internalization
MV20	P200S	near VP2/VP3 junction
	M469I	interior mutation
	A579T	surface exposed in VR-VIII/Loop 8 region, near sialic acid binding pocket and increases binding
MV50	G257R	surface region in VR-I/Loop1, increases binding and internalization
	S705G	surface mutation near known antigenic site
MV53	Q179R	near VP2/VP3 junction, increases binding, no effect on internalization
	G257R	surface region in VR-I/Loop1, increases binding and internalization

Location of the mutations was obtained by false color mapping of the mutations on to the crystal structure of the AAV5 capsid. Probable function was determined through structure analysis and binding/internalization assays.

mouse liver (Figure 4B). The only notable difference in tropism was a significant increase (4×) in LacZ expression levels in mouse lungs transduced by Loop1R variants when compared to WT-AAV5. (Figure 4C).

Assessing Neutralization of AAV5 Variants with Pre-existing Antibodies in IVIG

Next, we investigated whether directed evolution of AAV5 also induced changes in seroreactivity of the capsids to neutralizing factors in pooled human immunoglobulin G (IgG). Seroreactivity was assessed via a neutralizing assay that utilized intravenous IgG (IVIG), or pooled IgG from thousands of donors, to represent the overall prevalence of neutralizing antibodies against specific antigens in the overall human population. The mutant viruses were compared to two control serotypes, AAV5 and AAV9: one with very low prevalence of neutralizing antibodies (3%), and one with a medium prevalence (33.5%).[11] Curve fitting of the transformed and normalized data from the IVIG neutralization assay allowed for the comparison of seroreactivity between different viruses. From these curves, it was determined that the seroreactivity of AAV5 and the MV mutants are fairly consistent with each other; all of which are significantly shifted to the left versus AAV9, indicating that AAV5 and the mutants are less seroreactive and require more IVIG to neutralize their infectivity in comparison to AAV9 (Figure 5A). The calculated reciprocal dilution needed for 50% of inhibition for MV18 was significantly lower than AAV5, signifying that the Loop1R mutation slightly decreases seroreactivity of the AAV5 capsid (Figure 5B). MV20 and MV53 are slightly more seroreactive when compared to AAV5, whereas MV1 and MV50 do not differ in seroreactivity versus AAV5 (Figure 5B).

Elucidating Potential Mechanisms for AAV5 Mutations

The MV viruses were then investigated for changes in binding and internalization to understand how each mutation in each variant contributed to enhancing human liver tropism. First, the differences between MV18 and AAV5 were probed to determine effect of the G257R mutation. Binding assays revealed that the G257R mutation increased binding to the cell surface by a factor of 2.2 when compared to WT-AAV5 (Figure 6A). An internalization assay demonstrated the Loop1R mutation also increased the percentage of bound virions that were internalized by 3.1-fold versus WT-AAV5 (Figure 6C). The internalization assay additionally indicated a 7-fold difference in total virus internalized between AAV5 and the MV18, which was consistent with the difference in GFP expression reported previously (Figure 6B).

Subsequent comparison of MV18 against MV1, MV50, and MV53 allowed isolation of the effects of the secondary mutations and provided more insight into their potential functions. Binding and internalization assays corroborated the theory that the F417L mutation in MV1 affected a process post-internalization as there were no significant differences in binding or internalization between the MV1 and MV18 capsids (Figures 6D–6F). The S705G mutation in MV50 was found to increase binding to the cell surface around 2-fold over the Loop1R mutation alone (Figure 6D). The percentage of bound virions that were internalized decreased significantly when comparing MV50 to MV18, indicating that increased binding provided by S705G did not result in an increased percentage of internalized virus (Figure 6F). Similar results were found for MV53, where the Q179R mutation increased binding to the cell, but decreased the percentage of bound virions that were internalized (Figures 6D and 6F). No difference in the overall amount of internalized virus was observed between MV50, MV53, and MV18, signaling that the Loop1R mutation was dominant factor in the increase of internalization despite the increases in binding provided by the Q179R and S705G mutations (Figure 6E). Finally, the only mutant without the Loop1R mutation, MV20, exhibited binding comparable to MV18. However, the A579T mutation did not significantly increase the percentage of bound virions that are internalized when compared to AAV5 (Figures 6A and 6C).

DISCUSSION

Experience from clinical trials and literature has detailed the importance of balancing infectivity and immunogenicity when developing capsids for clinical use, increasing the complexity of the capsid engineering process.[16] To this end, many different methods have been employed to develop capsids with favorable seroreactivity that typically involve mutagenesis or evolution to diminish the seroreactivity of parental AAV serotypes.[8,26,27] We decided to approach the problem of balancing infectivity and seroprevalence through an alternative method that utilized random mutagenesis to engineer increased infectivity into AAV5, a serotype with poor liver infectivity, but extremely favorable seroreactivity.[9,11,12]

To accomplish this, a randomly mutated AAV5 capsid library was screened in Huh7 cells to select for AAV5 mutants with increased liver infectivity. After seven rounds of selection, two molecularly

Please cite this article in press as: Qian et al., Directed Evolution of AAV Serotype 5 for Increased Hepatocyte Transduction and Retained Low Humoral Seroreactivity, Molecular Therapy: Methods & Clinical Development (2020), https://doi.org/10.1016/j.omtm.2020.10.010

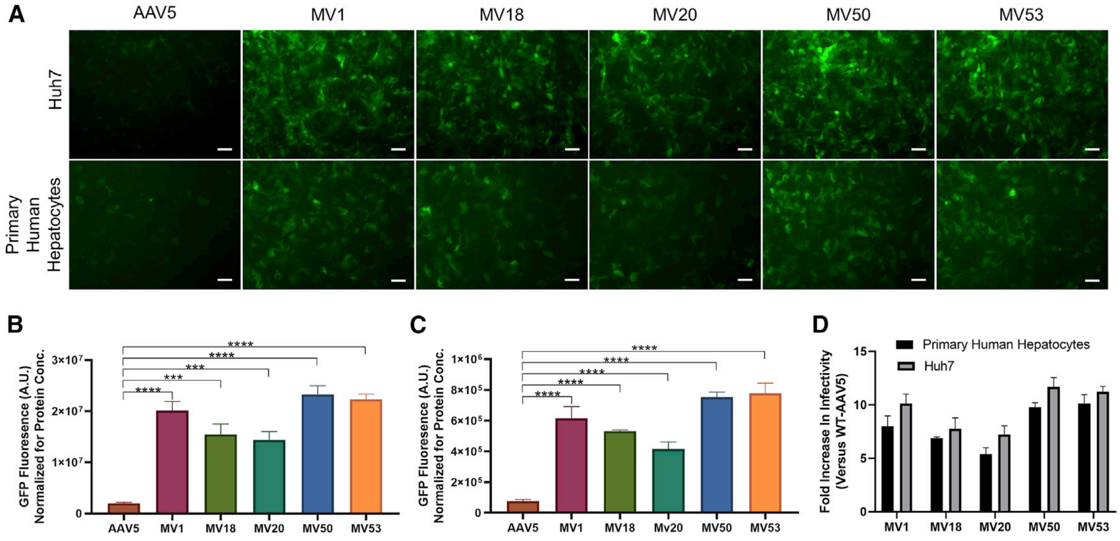

Figure 3. Evaluation of Huh7 and Primary Human Hepatocyte Transduction for Select AAV5 Mutants

(A) Fluorescent imaging of Huh7 cells and primary human hepatocytes (TRL HUM4037) transduced with self-complementary GFP vectors with AAV5 and MV mutant capsid serotypes. Huh7 cells were transduced at a MOI of 1e5 vg/cell and human hepatocytes were transduced at a MOI of 5e5vg/cell. Images were taken 72 (Huh7) and 96 (Hepatocytes) h after infection. Expression of GFP (green) indicates transduced cells. 10× magnification. Scale bar, 100 μm. (B) Comparison of normalized GFP expression in Huh7 cells between AAV5 and MV mutants. Data are shown as mean values ± SD. ***$p < 0.001$; ****$p < 0.0001$. (C) Comparison of normalized GFP expression in primary human hepatocytes between AAV5 and MV mutants. Data are shown as mean values ± SD. ****$p < 0.0001$. (D) Comparison of fold increases in GFP expression of MV mutants over WT-AAV5 in both Huh7 cells and primary human hepatocytes. Data are shown as mean values ± SD.

evolved AAV5 variants, MV50 and MV53, displayed significantly increased transduction in primary human hepatocytes by roughly 10-fold over WT-AAV5. MV50 and MV53 demonstrated that two single amino acid mutations in the AAV5 VP1 protein could significantly increase human hepatocyte transduction without substantially increasing seroreactivity. The MV mutants and AAV5 possess similar seroreactivities to IVIG, indicating that their seroprevalences in the human population are likely the same. This is largely because all the mutations found from the selection process are all *de novo* mutations and do not exist in any other serotype (Figure S3). Coupled with the fact that AAV5 is the most genetically divergent AAV serotype, it is extremely unlikely for any human to have been infected previously by these mutants and thus have developed neutralizing antibodies specifically targeting these *de novo* mutations.[17] Therefore the overall prevalence of neutralizing factors in the human population against the MV mutants is likely to be very similar to AAV5.

Non-humanized mouse transduction studies unsurprisingly revealed that the AAV5 variants with increased human liver transduction did not drastically show increased transduction in mouse liver, a phenomena that has been previously reported before.[9] Some variants even displayed de-targeting from mouse liver, which is again consistent with results from studies involving the engineering of human liver specific AAV capsids.[7,8] The secondary mutations in MV1 and MV53 could functional independently of species, as both enhanced liver transduction in human and mouse liver cells. The increased lung transduction of the MV mutants has potential applications but requires further validation on human lung cells.

Analysis utilizing binding, internalization, and location within the AAV5 capsid have shed some light on potential functions for each of the mutations. Our results demonstrated that the G257R mutation in the VR-I region of AAV5 is the primary driver of enhanced liver transduction and is likely increasing binding to a receptor that internalizes quickly, thus resulting in a larger portion of bound virus being internalized. For most other AAV serotypes, the VR-I region was discovered to be critical for binding of the AAV receptor (AAVR).[28–31] However, AAV5 was found to be the only AAVR-dependent serotype that does not interact with AAVR via the VR-I region, leading to uncertainty about the exact function of the AAV5 VR-I.[31] The G257R mutation likely indicates that the VR-I of AAV5 still binds an unknown receptor which subsequently also leads to increases in other cell types *in vitro* (Figure S6). Swapping of the AAV5 VR-I region with VR-I sequences from other serotypes resulted in destabilization of the AAV5 capsid indicating the VR-I region of AAV5 is not easily mutable (Table S1). Only two replacement Loop1 sequences, AAV7 loop1 and Avian AAV loop1, allowed for packaging of DNA, but were non-infectious in 293 cells and mice (Table S1). Further studies on the function of the VR-I region of AAV5 would provide valuable data in determining which receptor the G257R mutation is enhancing binding to.

Analysis of the secondary mutations found in the Loop1R mutants provided valuable information on the functions of various residues in the AAV5 capsid that could inform further engineering of the MV mutants. Due to decreases in yield (Figure S4) and its location in the capsid, the F417 mutation is likely decreasing the stability of

Please cite this article in press as: Qian et al., Directed Evolution of AAV Serotype 5 for Increased Hepatocyte Transduction and Retained Low Humoral Seroreactivity, Molecular Therapy: Methods & Clinical Development (2020), https://doi.org/10.1016/j.omtm.2020.10.010

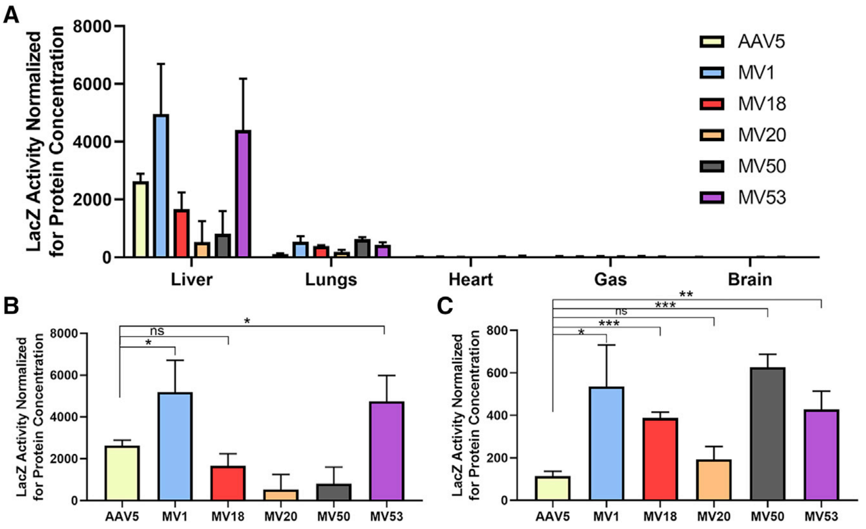

Figure 4. Comparison of Gene Transfer Efficiency in C57/BL6 Mice using a LacZ Vector

(A) Quantification of B-galactosidase (LacZ) activity in homogenized tissue samples of various organs in 8-week-old C57/Bl6 mice transduced by AAV5-CB-LacZnls, MV1-CB-LacZnls, MV18-CB-LacZnls, MV20-CB-LacZnls, MV50-CB-LacZnls, and MV53-CB-LacZnls. Each mouse was injected with 1e12 vg via tail-vein injection. Tissues were collected 4 weeks post injection. Tissue samples were homogenized and assayed for LacZ activity. LacZ activity was normalized by protein concentrations for each tissue sample. A total of five organs were quantitated for LacZ activity: the liver, lungs, heart, gastrocnemius (GAS), and brain. Data are shown as mean values ± SD. (B) Quantification of LacZ activity in homogenized mouse liver tissue. Data are shown as mean values ± SD. *p < 0.05. (C) Quantification of LacZ activity in homogenized mouse lung tissue. Data are shown as mean values ± SD. *p < 0.05. **p < 0.01. ***p < 0.001.

the capsid, which in turn could lead to more efficient uncoating. Uncoating has been reported to be the limiting step of gene expression in hepatocytes, and thus a potential increase in uncoating efficiency could lead to higher gene expression.[32] Studies have also shown AAV5 to be the most thermostable serotype, which prompts questions on whether its stability is negatively affecting uncoating.[33] A similar mutation to A579T in MV20 has been reported before in a study detailing a chimeric AAV5 mutant (A581T) with enhanced human lung transduction.[34,35] The A581T mutation was discovered to play a role in sialic acid recognition and binding, which could be a potential mechanism for the A579T mutation.[35] The S705G and Q179R mutations that are present in MV50 and MV53, respectively, seem to primarily affect binding to receptors that are not essential for internalization. The Q179R mutation is particularly interesting as it lies in the VP1/VP2 common region of the AAV5 VP1 protein, signifying that the presence of this mutation in a 60-mer capsid is significantly lower than a mutation that resides in the VP3 gene. Yet, the Q179R mutation is still able to significantly influence binding at comparable levels to the S705G mutation. The data presented in this study comparing MV18 and MV53 with binding and seroreactivity assays suggests that this particular region presides on or near the surface of the capsid, and also plays a role in binding and seroreactivity. This could have interesting implications on the understanding and engineering of AAV5 as there have been no other reports of binding domains found in the VP1/VP2 common region thus far for AAV5 or any other serotype. Combining mutations from the MV mutants did not further increase transduction efficacy (Figure S5), indicating that further improvement of these capsids may need to be achieved by additional directed evolution or targeted rational design of VR-VIII.

Another goal of the study was to demonstrate the translatability of AAV infectivity in Huh7 cells to primary human hepatocytes. Previous studies have alluded to the similarities of AAV transduction in Huh7 cells and human hepatocytes, but here in this study, we determined that Huh7 cells can serve as an adequate selection model for human hepatocyte transduction.[36] While many studies have opted for *in vivo* selection of AAV capsids, we believe that the fenestrated nature of the endothelial lining in the liver allows for easier translation of liver infectivity from *in vitro* to *in vivo*, as long as the screening system is of human hepatocyte origin.[37] Twenty percent of the surface of the liver sinusoidal endothelial lining is covered by fenestrations that allow nano-sized particles to pass from blood to hepatocytes, effectively allowing AAV to access hepatocytes without needing to first cross the endothelial barrier.[38,39] As such, screening for liver infectivity *in vitro* may not face the same barriers that others have noted when screening for muscle or CNS capsids.[40,41] Additionally, there is a wealth of evidence that supports the idea that non-humanized mouse models are not suited for screening of hepatotropic properties in human hepatocytes.[9,42] Even humanized liver mouse models present their own challenges, which include factors such as variability of the number of implanted human hepatocyte versus existing mouse hepatocytes and morphology of the humanized livers.[8,43] All of these factors indicated that screening for human liver transduction in an *in vivo* model was not the only route that could be taken and that *in vitro* selection could potentially yield capsids with increased human liver infectivity.

Our engineered AAV5 capsids could potentially be used to improve current hemophilia A and B therapies that utilize WT-AAV5 by increasing the expression levels of Factor VIII and XI at decreased doses. AAV5-based therapies typically require extremely high doses of AAV5, which also increases production cost of the treatments, rendering them less obtainable for potential patients. Our results demonstrated that AAV5 can be engineered to have increased human hepatocyte transduction while retaining its favorable seroreactivity and provided a roadmap for an alternative method of developing liver tropic AAV vectors from capsids with poor infectivity and low seroprevalence. However, for liver diseases requiring higher transduction

Please cite this article in press as: Qian et al., Directed Evolution of AAV Serotype 5 for Increased Hepatocyte Transduction and Retained Low Humoral Seroreactivity, Molecular Therapy: Methods & Clinical Development (2020), https://doi.org/10.1016/j.omtm.2020.10.010

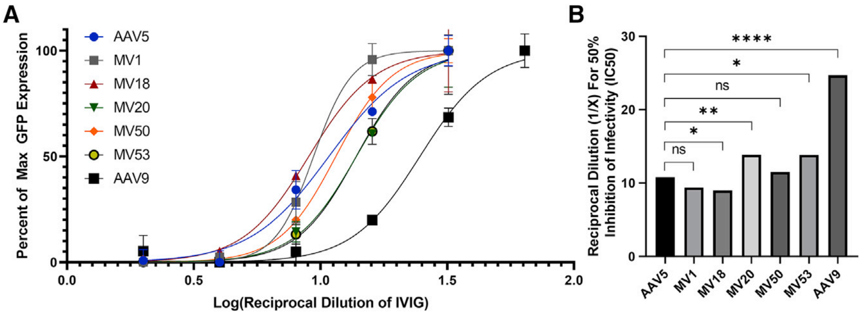

C

RECIPROCAL DILUTION	1	2	4	8	16	32	64
IVIG(UG)	25	12.5	6.25	3.13	1.56	0.78	0.39
VIRUS(VG)	1e9	1e9	1e9	1e9	1e9	1e9	1e9

Figure 5. Seroreactivity of AAV5 and MV Mutants using an IVIG-Based Assay

(A) AAV neutralization assay using pooled human immunoglobulins from thousands of donors (IVIG Carimune NF, ZLB Behring) to assess seroreactivity. MV mutants were compared to AAV5 and AAV9 in their ability to resist neutralization by IVIG. Each capsid serotype containing sc-GFP was incubated with reciprocal dilutions of IVIG and added to Huh7 cells. After 72 h, the GFP expression for each reciprocal dilution was quantified and compared to GFP expression of an infection control without the presence of IVIG. The percentage of max GFP expression at each reciprocal dilution for each virus was used to generate curves that represented the seroreactivity of each virus. Graphpad Prism 8 was used to curve fit the data. Serotypes with curves further to the right indicate less IVIG is needed to neutralize the virus. Serotypes with curves further to the left indicate more IVIG is needed to neutralize the virus. Plotted data points are shown as mean values ± SD. (B) Reciprocal dilution at which 50% inhibition is achieved was calculated from the best fit curves for each capsid. Extra sum of squares F test was used to compare the best fit curves for each capsid and determine whether one capsid is more/less seroreactive compared to AAV5. *$p < 0.05$; **$p < 0.01$; ****$p < 0.0001$. (C) Approximate amounts of IVIG and virus used for each reciprocal dilution.

levels, the MV capsids likely will not be sufficient and will need to be further modified via additional directed evolution. Further evolution of these mutants could also result in capsids that are even less seroreactive compared to WT-AAV5. For diseases requiring tropism to other areas such as the CNS or skeletal muscle, more drastic evolution of AAV5 is needed. In these cases, peptide insertion libraries, or targeted mutations of specific loops can be used, but more caution must be placed when determining the changes in seroreactivity.

MATERIALS AND METHODS

Generation of Random Mutagenesis AAV5 Plasmid Library

The randomly mutated AAV5 plasmid library was generated by amplifying a WT-AAV5 capsid gene (XR5 plasmid) with error prone PCR using a GeneMorph II Random Mutagenesis Kit (Agilent, Santa Clara, CA, USA) followed by subsequent amplification by stEP. In brief, 10 ng of the DNA template was amplified over 30 cycles using an error prone DNA polymerase to achieve a wide range in the VP3 gene per each PCR fragment. To further diversify the mutant AAV5 plasmid library, we shuffled the point mutations generated by error prone PCR using stEP in which roughly 100 ng of mutated AAV5 VP3 DNA was subjected to 100 extremely short cycles of denaturing (∼20 s), annealing (∼5 s), and elongation (∼10 s). For both error prone pcr and stEP, the forward primer (Sigma-Aldrich, St. Louis, MO, USA; 5′-AGGCTCGGACCGAAGAGGACT-3′) overlapped a RSRII restriction enzyme cutsite upstream of the VP3 gene while the reverse primer (Sigma-Aldrich, St. Louis, MO, USA; 5′-ATCGAGCGGCCGCAAGAGGCAGTATTTTAC-3′) overlapped the NotI cutsite downstream of the VP3 gene to facilitate cloning of the PCR product into an "infectious," or WT-AAV5 plasmid.

The mutant VP3 stEP PCR amplicons were purified and digested to completion using RSRII/NotI restriction enzymes (NEB, Ipswich, MA, USA). Digested DNA was isolated using agarose gel purification and cloned into backbone containing an AAV2 REP gene and a partial AAV5 Capsid gene flanked by AAV2 ITR sequences to create an "infectious" plasmid, or a replication competent AAV plasmid. The resulting mixture was electroporated into MAX efficiency^{tm} DH10B electrocompetent bacteria (Invitrogen, Carlsbad, CA, USA) and spread across LB agar plates containing ampicillin. A viral plasmid library of 5×10^5 independent clones was generated as determined by quantification of the number of colonies following bacteria transformation. To confirm the diversity of the library, we picked 20 colonies from the plates for miniprep and sequencing. Individual bacteria colonies were pooled together by washing the LB-agar plates with terrific broth medium and then cultured in four liters of TB medium. The propagated bacteria were then used for large-scale purification of the plasmid DNA library by ultracentrifugation with CsCl-ethidium bromide gradients.

AAV Library and Vector Production

Purified AAV vectors were produced via the triple transfection and cesium chloride density ultracentrifugation methods. Briefly, HEK293 cells were transfected with a helper plasmid, a self-complementary GFP (sc-GFP) or single stranded LacZ-nls vector plasmid, and a packaging plasmid containing capsid gene and an AAV2 rep gene. Cells were harvested 72 h post transfection and isolated by centrifugation. The media was collected and stored while the cell pellet was resuspended and subjected to three freeze thaw cycles. The resulting cell lysate was incubated with DnaseI and RnaseA at 37°C and centrifuged and the supernatant was collected. The virus from the supernatant and the media were then concentrated by PEG precipitation followed by two rounds of cesium chloride (CsCl) density ultracentrifugation. CsCl fractions containing virus were determined by DNA dotblot and combined and dialyzed using 5% sorbitol in PBS. Dialyzed virus preparations were again titered using DNA dot blot with a probe specific to reporter gene. All viruses that were tested were titered in the same dotblot to allow consistent measurement of viral titers.

Please cite this article in press as: Qian et al., Directed Evolution of AAV Serotype 5 for Increased Hepatocyte Transduction and Retained Low Humoral Seroreactivity, Molecular Therapy: Methods & Clinical Development (2020), https://doi.org/10.1016/j.omtm.2020.10.010

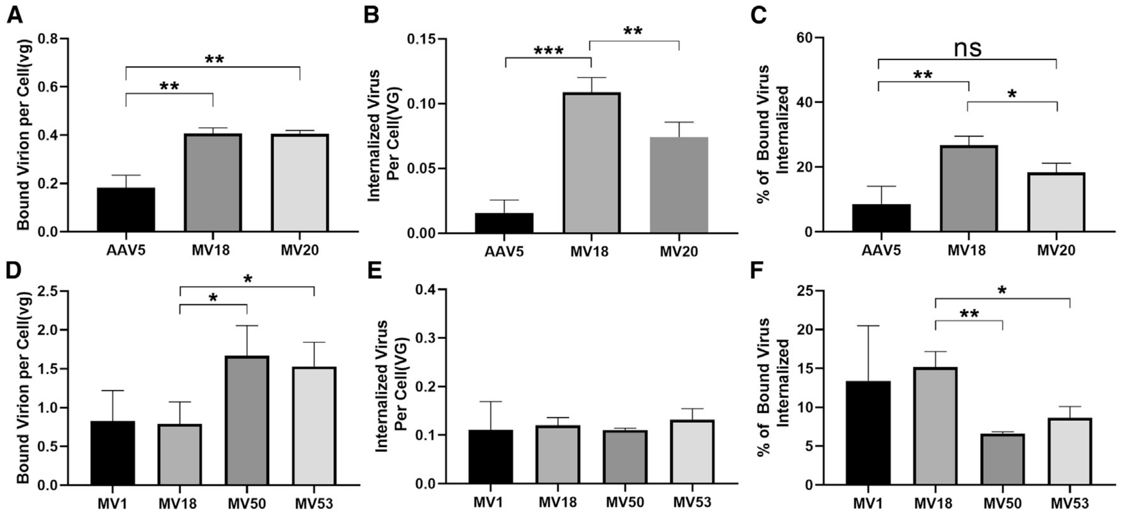

Figure 6. Comparison of Binding and Internalization between AAV5 and MV Mutants

(A) Binding assay comparing binding of capsid to Huh7 cell surface between AAV5, MV18, and MV20. Data shown are mean values ± SD. $^{**}p < 0.01$. (B) Internalization assay comparing amount of internalized virus in Huh7 cells between AAV5, MV18, and MV20. Data shown are mean values ± SD. $^{**}p < 0.01$; $^{***}p < 0.001$. (C) Percentage of bound viral genomes that are internalized comparison between AAV5, MV18, and MV20. Data shown are mean values ± SD. $^{*}p < 0.05$; $^{**}p < 0.01$. (D) Binding assay comparing
Q10 binding of capsid to Huh7 cell surface between MV1, MV18, MV50, and MV53. Data shown are mean values ± SD. $^{**}p < 0.01$. (E) Internalization assay comparing amount of internalized virus in Huh7 cells between MV1, MV18, MV50, and MV53. Data shown are mean values ± SD. (F) Percentage of bound viral genomes that are internalized comparison between MV1, MV18, MV50, and MV53. Data shown are mean values ± SD. $^{*}p < 0.05$; $^{**}p < 0.01$.

The AAV5 viral library was produced using the same method as described before, but with only two plasmids. In brief, the plasmid library and a helper plasmid were transfected into human embryonic kidney (HEK293) cells at a 1:1 ratio via the calcium phosphate transfection method. After 72 h post transfection, the transfected cells and media were collected. The viral particles were released via the freeze thaw method and purified using CsCl density centrifugation. For all AAV5 library experiments, the AAV titer was determined by the dot-blot method using a probe specific to the AAV2 Rep gene.

Mutant AAV5 Library Selection in Huh7 Cells

The AAV5 viral library was added to Huh7 cells at a MOI of 5,000 vector genomes per cell and coinfected with WT-adenovirus type-5 (WT-ad) at a MOI of 100 vg per cell. After 72 h of infection, the cells were collected and lysed via three freeze thaw cycles with a dry ice and ethanol mixture. The cell lysate was then incubated at 56°C for 1 h to inactivate the WT-ad present in lysate. The cell lysate was then centrifuged at 15,000 rpm for 5 min and the supernatant containing the mutant viral particles was collected. The virus in the supernatant was titered by DNA dotblot. Huh7 cells were again infected with virus from the previous round of selection at a MOI of 5,000 vg/cell. In total, 7 rounds of selection on Huh7 cells were performed. For the last round of selection, the infected cells were washed with PBS twice to remove any virus that did not manage to infect the cells. The viral DNA was then extracted from the cells via a modified Hirt Extraction as previously described by others.[44] In short, cells were suspended in 250 μL of a Tris-HCl/EDTA solution containing 100 μg/mL RNase A. Cells were lysed with the addition of a 1.2% sodium dodecyl sulfate solution and incubated for 5 min at room temperature. Chromosomal DNA and cellular debris were precipitated with the addition of a cesium chloride, potassium acetate, and acetic acid solution and incubated on ice for 15 min. The mixture was then centrifuged at 4°C for 15 min, and the supernatant was loaded onto a QIAGEN Qiaprep Spin column (QIAGEN, Hilden, Germany). The bound DNA is then washed, eluted and amplified via PCR using the same primers used for the error prone PCR. The resulting PCR amplicons are purified and subsequently digested with RSRII and NotI and ligated into a packaging plasmid containing AAV2 Rep and a partial AAV5 Cap gene. The ligation mixture was transformed into bacteria and spread onto agar plates containing ampicillin. After overnight incubation, individual colonies were picked for plasmid amplification via miniprep. Amplified plasmid DNA was incubated with RNASEA, purified, and sequenced. Primers used to sequence mutant AAV5 capsids were: F1: 5′-GGCCAGGCTCTCATTTGTTC-3′; F2: 5′-GACGACACATCC TTCGGG-3′; F3: 5′-CAGCTGCCCTACGTCGTC-3′; F4: 5′-GG CACGTACAACCTCCAGGAAATC-3′

Sequence and Structure Analysis of AAV5 Variant Capsid Genes

DNA and protein sequences of mutant AAV5 capsid genes screened in Huh7 cells were identified and aligned with Sequencher, AlignX, and Clustal X. Amino acid mutations for each variant were false-color mapped on to the AAV5 VP3 protein structure 3NTT using PyMol v2.3.0. 60-mer AAV5 capsid structures were obtained by using 3NTT and viperdb to generate a 60-mer consisting of 60 AAV5 VP3 proteins. The mutations from variant AAV5 capsids were false color mapped onto the 60-mer structure.

Please cite this article in press as: Qian et al., Directed Evolution of AAV Serotype 5 for Increased Hepatocyte Transduction and Retained Low Humoral Seroreactivity, Molecular Therapy: Methods & Clinical Development (2020), https://doi.org/10.1016/j.omtm.2020.10.010

Transduction of Human Liver Cancer Cells by AAV Vectors

Variant AAV5 vectors were used to packaged sc-GFP reporter genes for comparison to WT-AAV5. Huh7 cells in 12 well plates were infected with AAV5 and mutant self-complementary (sc)-GFP encoding viruses at a MOI of 100,000 viral genomes (vg) per cell and co-infected with 5,000 vg/cell of WT-ad5. 72 h after infection, cells were taken for fluorescent imaging and subsequent GFP quantification using the Fluorometric GFP Quantification Kit (Cell BioLabs, San Diego, CA, USA). GFP activity was normalized by protein concentration as measured by Pierce BCA Protein Assay Kit (Thermo Fisher Scientific, Waltham, MA, USA).

Transduction of Primary Human Hepatocytes by AAV Vectors

Primary human hepatocytes (TRL HUM4037 and HUM4020, Triangle Research Labs, Durham, NC, USA) were cultured according to vendor's instructions. In brief, cells were thawed and plated at 2.5e5 cells per well in collagen coated 24 well plates (BioCoat Collagen I 24 well plates, Corning, Corning, NY, USA). The next day, the primary human hepatocytes were infected with purified sc-GFP vectors at a MOI of 500,000 vg per cell. Media was changed 48 h after infection, and once daily afterward. 96 h post infection, cells were taken for fluorescent imaging and subsequent GFP quantification using the Fluorometric GFP Quantification Kit from Cell Bio Labs (San Diego, CA, USA). GFP activity was normalized by protein concentration as measured by Pierce BCA Protein Assay Kit (Thermo Fisher Scientific, Waltham, MA, USA).

Tissue Tropism of AAV Vectors in Mice after Systemic Administration

C57/BL6J mice were maintained in a 12-h:12-h light:dark artificial light Q9 cycle (07:00–19:00) at a temperature of 20°C and a humidity of 55% ± 5%. All animal protocols were approved by the University of North Carolina Animal Care and Use Committee. The mutant AAV5 capsid genes were used to package CB-LacZnls reporter gene, which expresses B-galactosidase with a nuclear localization signal and compared to WT-AAV5. LacZ vectors were administered to 8-week-old male C57BL/6J mice by intravenous tail vein injection at a dose of 1e12 vg per mouse. After 3 weeks, the mice were sacrificed, and tissues were collected, and flash frozen in liquid nitrogen cooled 2-methylbutane. Tissues were stored at −80°C until ready for use. Tissues were homogenized in lysis buffer from the Galacto-Star LacZ Assay Kit (Applied BioSystems, Foster City, CA, USA) and measured for protein concentration using the Pierce BCA Protein Assay Kit (Thermo Fisher, Waltham, MA, USA). Samples were diluted to similar protein concentrations. B-galactosidase enzyme activity in homogenized tissue lysates was measured using the Galacto-Star LacZ Assay Kit and readings were normalized by protein concentration.

AAV Neutralization Assays with IVIG

Huh7 cells were seeded at 5e4 cells per well in a 96 well plate. The next day, 50 particles of WT-ad5 per cell was added to each well of the 96 well plate. AAV sc-GFP vectors (1e9 vg) were incubated with reciprocal dilutions of IVIG (Carimune NF, ZLB Behring, King of Prussia, PA, USA). The first well corresponded to 25 μg of IVIG, and each subsequent well contained half of the previous amount of IVIG. The combined IVIG and AAV vector mixtures were incubated for 1 h at 37°C and then added to individual wells of Huh7 cells in a 96 well plate. Virus concentration was kept the same for each reciprocal dilution of IVIG. After 72 h, the cells in each well were lysed and the crude lysate was assayed for GFP activity using a Fluorometric GFP Quantification Kit (CellBio Labs, San Diego, CA, USA). The GFP activity was measured against a control well infected with only virus and no IVIG to obtain the percent of max GFP expression at each reciprocal dilution for each virus. The percent of max GFP activity was plotted against the log reciprocal dilution in GraphPad 8 to generate a fitted curve using nonlinear regression. The corresponding EC_{50}, or reciprocal dilution required for 50% max GFP activity, was calculated using GraphPad 8, and compared to the EC_{50} of other AAV serotypes to determine the relative differences in seroreactivity to IVIG.

Binding and Internalization Assay of AAV Vectors in Huh7 Cells

Binding of the AAV capsid to the surface of Huh7 cells was determined by incubating 2e3vg/cell of CB-LacZnls AAV vector with pre-cooled Huh7 Cells at 4°C for 30 min. Then, the cells were washed with cold PBS three times to wash away any unbound vector. Cells were collected and resuspended in PBS. Total DNA was extracted using the DNeasy Blood and Tissue Kit (QIAGEN, Hilden, Germany) and eluted with molecular grade water. The number of vector copies was determined in each sample via absolute qPCR quantification using primers and TaqMan probe specifically recognizing the CB-promoter sequence (Sigma Aldrich, St. Louis, MO, USA). The number of vector genomes was normalized to the number of endogenous glucagon gene copies. Internalization assay was performed similar to the binding assay, but instead allowing the bound virus to internalize at 37°C for 1 h. Cells were incubated with trypsin for 5 min at 37°C to allow any un-internalized virus to be digested. After washing with PBS three times, total DNA was extracted from cells and eluted with molecular grade water. Vector copies was determined exactly the same as described before. Assays were performed on a 7300 Real-Time PCR system (Applied Biosystems) using GoTaq PCR Master Mix (Promega). The primers and probes used are as follows: CB-F: 5′-GTATGTTCCCATAGTAACGCCAATAG-3′; CB-R: 5′-GGCGTACTTGGCATATGATACACT-3′; CB Probe: 5′-FAM-TCAATGGGTGGAGTATTTA-MGB-3′; Glucagon-F: 5′-AAGGGA CCTTTACCAGTGATGTG-3′; Glucagon-R: 5′-ACTTACTCT CGCCTTCCTCGG-3′; Glucagon Probe: 5′-FAMCAGCAAAGGAA TTCA-MGB-3′.

Site-Directed Mutagenesis for Combining Mutations from Different Variants

Mutations were combined onto one capsid gene using site directed mutagenesis. A primer containing the mutation was used with a primer that covered half the ampicillin gene to amplify half of the MV mutant packaging plasmid. A primer set corresponding to the other half of the plasmid was also used to amplify the opposing half of the plasmid. The two PCR amplicons were purified and digested with DPNI (NEB, Ipswich, MA, USA) and purified using gel purification via QIAquick Gel Extraction Kit (QIAGEN, Hilden, Germany).

Please cite this article in press as: Qian et al., Directed Evolution of AAV Serotype 5 for Increased Hepatocyte Transduction and Retained Low Humoral Seroreactivity, Molecular Therapy: Methods & Clinical Development (2020), https://doi.org/10.1016/j.omtm.2020.10.010

The purified fragments were ligated together using blunt end ligation and transformed into DH10B competent cells. Transformed bacteria was then spread on an agar plate and allowed to grow overnight. Individual colonies were picked for DNA amplification and extraction. Extracted plasmids were then sent for sequencing to confirm proper incorporation of the mutations.

The primers used are as follows: F417L MV1-F: 5′-TTCCACTCCAGCCTCGCTCCCAGT-3′; F417L MV1-R: 5′-GGGCACCTCCTCAAAGTTGTA-3′; A579T MV20-F: 5′-GACCGGCACGTACAACCTCCA-3′; A579T MV20-R: 5′-GCGGGGGTAGTGGTGGAGC-3′; S705G MV50-F: 5′-CACCGGGGAATACAGAAGCACC-3′; S705G MV50-R: 5′-CCGTCCGGGGCAAAGTCCAC-3′; Q179R MV53-F: 5′-CTGCGAATCCCAGCCCAACCA-3′; Q179R MV53-R: 5′-CTGCTGGGATCCGCTGGGTC-3′; AMP-R: 5′-ACTCACCAGTCACAGAAAAGCATC-3′; AMP-F: 5′-ACTCAACCAAGTCATTCTGAGAATAG-3′.

Statistics

Statistical analyses were conducted with GraphPad Prism 8. All statistical analyses were justified as appropriate and p values <0.05 were considered statistically significant. For all statistical tests performed, intra-assay variation fell within the expected range and the variance across groups was similar. Comparison of experimental values from two groups were assessed using a Student's unpaired two-tailed t test. Experimental values for Figure 5A were first normalized and then plotted against the log transformed values for reciprocal dilutions before being compared using an extra sum of squares F test.

SUPPLEMENTAL INFORMATION

Supplemental Information can be found online at https://doi.org/10.1016/j.omtm.2020.10.010.

AUTHOR CONTRIBUTIONS

R.Q., B.X., J.L., and X.X. designed the experiments. R.Q. carried out the experiments. R.Q. performed analysis of the data. R.Q., B.X., J.L., and X.X. contributed to interpretation of the results. R.Q. prepared the manuscript and figures. X.X. secured funding acquisition. All authors reviewed and provided critical feedback on the final manuscript.

CONFLICTS OF INTEREST

X.X., J.L., and R.Q. are co-inventors on a patent application for AAV serotypes generated in this paper.

ACKNOWLEDGMENTS

Q11 This work was supported by an award from the Eshelman Institute of Innovation (EII) to X.X.

REFERENCES

1. George, L.A., Sullivan, S.K., Giermasz, A., Rasko, J.E.J., Samelson-Jones, B.J., Ducore, J., Cuker, A., Sullivan, L.M., Majumdar, S., Teitel, J., et al. (2017). Hemophilia B Gene Therapy with a High-Specific-Activity Factor IX Variant. N. Engl. J. Med. *377*, 2215–2227.
2. Pasi, K.J., Rangarajan, S., Mitchell, N., Lester, W., Symington, E., Madan, B., Laffan, M., Russell, C.B., Li, M., Pierce, G.F., and Wong, W.Y. (2020). Multiyear Follow-up of AAV5-hFVIII-SQ Gene Therapy for Hemophilia A. N. Engl. J. Med. *382*, 29–40.
3. Rangarajan, S., Walsh, L., Lester, W., Perry, D., Madan, B., Laffan, M., Yu, H., Vettermann, C., Pierce, G.F., Wong, W.Y., and Pasi, K.J. (2017). AAV5-Factor VIII Gene Transfer in Severe Hemophilia A. N. Engl. J. Med. *377*, 2519–2530.
4. Colella, P., Ronzitti, G., and Mingozzi, F. (2017). Emerging Issues in AAV-Mediated *In Vivo* Gene Therapy. Mol. Ther. Methods Clin. Dev. *8*, 87–104.
5. Doshi, B.S., and Arruda, V.R. (2018). Gene therapy for hemophilia: what does the future hold? Ther. Adv. Hematol. *9*, 273–293.
6. Kattenhorn, L.M., Tipper, C.H., Stoica, L., Geraghty, D.S., Wright, T.L., Clark, K.R., and Wadsworth, S.C. (2016). Adeno-Associated Virus Gene Therapy for Liver Disease. Hum. Gene Ther. *27*, 947–961.
7. Lisowski, L., Dane, A.P., Chu, K., Zhang, Y., Cunningham, S.C., Wilson, E.M., Nygaard, S., Grompe, M., Alexander, I.E., and Kay, M.A. (2014). Selection and evaluation of clinically relevant AAV variants in a xenograft liver model. Nature *506*, 382–386.
8. Paulk, N.K., Pekrun, K., Zhu, E., Nygaard, S., Li, B., Xu, J., Chu, K., Leborgne, C., Dane, A.P., Haft, A., et al. (2018). Bioengineered AAV Capsids with Combined High Human Liver Transduction In Vivo and Unique Humoral Seroreactivity. Mol. Ther. *26*, 289–303.
9. Vercauteren, K., Hoffman, B.E., Zolotukhin, I., Keeler, G.D., Xiao, J.W., Basner-Tschakarjan, E., High, K.A., Ertl, H.C., Rice, C.M., Srivastava, A., et al. (2016). Superior In vivo Transduction of Human Hepatocytes Using Engineered AAV3 Capsid. Mol. Ther. *24*, 1042–1049.
10. Perocheau, D.P., Cunningham, S., Lee, J., Antinao Diaz, J., Waddington, S.N., Gilmour, K., Eaglestone, S., Lisowski, L., Thrasher, A.J., Alexander, I.E., et al. (2019). Age-Related Seroprevalence of Antibodies Against AAV-LK03 in a UK Population Cohort. Hum. Gene Ther. *30*, 79–87.
11. Boutin, S., Monteilhet, V., Veron, P., Leborgne, C., Benveniste, O., Montus, M.F., and Masurier, C. (2010). Prevalence of serum IgG and neutralizing factors against adeno-associated virus (AAV) types 1, 2, 5, 6, 8, and 9 in the healthy population: implications for gene therapy using AAV vectors. Hum. Gene Ther. *21*, 704–712.
12. Halbert, C.L., Miller, A.D., McNamara, S., Emerson, J., Gibson, R.L., Ramsey, B., and Aitken, M.L. (2006). Prevalence of neutralizing antibodies against adeno-associated virus (AAV) types 2, 5, and 6 in cystic fibrosis and normal populations: Implications for gene therapy using AAV vectors. Hum. Gene Ther. *17*, 440–447.
13. Ling, C., Wang, Y., Feng, Y.L., Zhang, Y.N., Li, J., Hu, X.R., Wang, L.N., Zhong, M.F., Zhai, X.F., Zolotukhin, I., et al. (2015). Prevalence of neutralizing antibodies against liver-tropic adeno-associated virus serotype vectors in 100 healthy Chinese and its potential relation to body constitutions. J. Integr. Med. *13*, 341–346.
14. Calcedo, R., and Wilson, J.M. (2016). AAV Natural Infection Induces Broad Cross-Neutralizing Antibody Responses to Multiple AAV Serotypes in Chimpanzees. Hum. Gene Ther. Clin. Dev. *27*, 79–82.
15. High, K.A., and Aubourg, P. (2011). rAAV human trial experience. Methods Mol. Biol. *807*, 429–457.
16. Mingozzi, F., and High, K.A. (2013). Immune responses to AAV vectors: overcoming barriers to successful gene therapy. Blood *122*, 23–36.
17. Bantel-Schaal, U., Delius, H., Schmidt, R., and zur Hausen, H. (1999). Human adeno-associated virus type 5 is only distantly related to other known primate helper-dependent parvoviruses. J. Virol. *73*, 939–947.
18. Stanford, S., Pink, R., Creagh, D., Clark, A., Lowe, G., Curry, N., Pasi, J., Perry, D., Fong, S., Hayes, G., et al. (2019). Adenovirus-associated antibodies in UK cohort of hemophilia patients: A seroprevalence study of the presence of adenovirus-associated virus vector-serotypes AAV5 and AAV8 neutralizing activity and antibodies in patients with hemophilia A. Res. Pract. Thromb. Haemost. *3*, 261–267.
19. Majowicz, A., Nijmeijer, B., Lampen, M.H., Spronck, L., de Haan, M., Petry, H., van Deventer, S.J., Meyer, C., Tangelder, M., and Ferreira, V. (2019). Therapeutic hFIX Activity Achieved after Single AAV5-hFIX Treatment in Hemophilia B Patients and NHPs with Pre-existing Anti-AAV5 NABs. Mol. Ther. Methods Clin. Dev. *14*, 27–36.

20. Zhao, H., Giver, L., Shao, Z., Affholter, J.A., and Arnold, F.H. (1998). Molecular evolution by staggered extension process (StEP) in vitro recombination. Nat. Biotechnol. *16*, 258–261.
21. Schaffer, D.V., and Maheshri, N. (2004). Directed evolution of AAV mutants for enhanced gene delivery. Conf. Proc. IEEE Eng. Med. Biol. Soc. *2004*, 3520–3523.
22. Maheshri, N., Koerber, J.T., Kaspar, B.K., and Schaffer, D.V. (2006). Directed evolution of adeno-associated virus yields enhanced gene delivery vectors. Nat. Biotechnol. *24*, 198–204.
23. Tseng, Y.S., and Agbandje-McKenna, M. (2014). Mapping the AAV Capsid Host Antibody Response toward the Development of Second Generation Gene Delivery Vectors. Front. Immunol. *5*, 9.
24. Walters, R.W., Agbandje-McKenna, M., Bowman, V.D., Moninger, T.O., Olson, N.H., Seiler, M., Chiorini, J.A., Baker, T.S., and Zabner, J. (2004). Structure of adeno-associated virus serotype 5. J. Virol. *78*, 3361–3371.
25. Afione, S., DiMattia, M.A., Halder, S., Di Pasquale, G., Agbandje-McKenna, M., and Chiorini, J.A. (2015). Identification and mutagenesis of the adeno-associated virus 5 sialic acid binding region. J. Virol. *89*, 1660–1672.
26. Tse, L.V., Klinc, K.A., Madigan, V.J., Castellanos Rivera, R.M., Wells, L.F., Havlik, L.P., Smith, J.K., Agbandje-McKenna, M., and Asokan, A. (2017). Structure-guided evolution of antigenically distinct adeno-associated virus variants for immune evasion. Proc. Natl. Acad. Sci. USA *114*, E4812–E4821.
27. Grimm, D., Lee, J.S., Wang, L., Desai, T., Akache, B., Storm, T.A., and Kay, M.A. (2008). In vitro and in vivo gene therapy vector evolution via multispecies interbreeding and retargeting of adeno-associated viruses. J. Virol. *82*, 5887–5911.
28. Dudek, A.M., Pillay, S., Puschnik, A.S., Nagamine, C.M., Cheng, F., Qiu, J., Carette, J.E., and Vandenberghe, L.H. (2018). An Alternate Route for Adeno-associated Virus (AAV) Entry Independent of AAV Receptor. J. Virol. *92*, e02213-17.
29. Meyer, N.L., Hu, G., Davulcu, O., Xie, Q., Noble, A.J., Yoshioka, C., Gingerich, D.S., Trzynka, A., David, L., Stagg, S.M., and Chapman, M.S. (2019). Structure of the gene therapy vector, adeno-associated virus with its cell receptor, AAVR. eLife *8*, e44707.
30. Zhang, R., Cao, L., Cui, M., Sun, Z., Hu, M., Zhang, R., Stuart, W., Zhao, X., Yang, Z., Li, X., et al. (2019). Adeno-associated virus 2 bound to its cellular receptor AAVR. Nat. Microbiol. *4*, 675–682.
31. Zhang, R., Xu, G., Cao, L., Sun, Z., He, Y., Cui, M., Sun, Y., Li, S., Li, H., Qin, L., et al. (2019). Divergent engagements between adeno-associated viruses with their cellular receptor AAVR. Nat. Commun. *10*, 3760.
32. Thomas, C.E., Storm, T.A., Huang, Z., and Kay, M.A. (2004). Rapid uncoating of vector genomes is the key to efficient liver transduction with pseudotyped adeno-associated virus vectors. J. Virol. *78*, 3110–3122.
33. Bennett, A., Patel, S., Mietzsch, M., Jose, A., Lins-Austin, B., Yu, J.C., Bothner, B., McKenna, R., and Agbandje-McKenna, M. (2017). Thermal Stability as a Determinant of AAV Serotype Identity. Mol. Ther. Methods Clin. Dev. *6*, 171–182.
34. Excoffon, K.J., Koerber, J.T., Dickey, D.D., Murtha, M., Keshavjee, S., Kaspar, B.K., Zabner, J., and Schaffer, D.V. (2009). Directed evolution of adeno-associated virus to an infectious respiratory virus. Proc. Natl. Acad. Sci. USA *106*, 3865–3870.
35. Dickey, D.D., Excoffon, K.J., Koerber, J.T., Bergen, J., Steines, B., Klesney-Tait, J., Schaffer, D.V., and Zabner, J. (2011). Enhanced sialic acid-dependent endocytosis explains the increased efficiency of infection of airway epithelia by a novel adeno-associated virus. J. Virol. *85*, 9023–9030.
36. Li, S., Ling, C., Zhong, L., Li, M., Su, Q., He, R., Tang, Q., Greiner, D.L., Shultz, L.D., Brehm, M.A., et al. (2015). Efficient and Targeted Transduction of Nonhuman Primate Liver With Systemically Delivered Optimized AAV3B Vectors. Mol. Ther. *23*, 1867–1876.
37. Braet, F., and Wisse, E. (2002). Structural and functional aspects of liver sinusoidal endothelial cell fenestrae: a review. Comp. Hepatol. *1*, 1.
38. Cogger, V.C., O'Reilly, J.N., Warren, A., and Le Couteur, D.G. (2015). A standardized method for the analysis of liver sinusoidal endothelial cells and their fenestrations by scanning electron microscopy. J. Vis. Exp. *30*, e52698.
39. Wisse, E., De Zanger, R.B., Charels, K., Van Der Smissen, P., and McCuskey, R.S. (1985). The liver sieve: considerations concerning the structure and function of endothelial fenestrae, the sinusoidal wall and the space of Disse. Hepatology *5*, 683–692.
40. Deverman, B.E., Pravdo, P.L., Simpson, B.P., Kumar, S.R., Chan, K.Y., Banerjee, A., Wu, W.L., Yang, B., Huber, N., Pasca, S.P., and Gradinaru, V. (2016). Cre-dependent selection yields AAV variants for widespread gene transfer to the adult brain. Nat. Biotechnol. *34*, 204–209.
41. Yang, L., Jiang, J., Drouin, L.M., Agbandje-McKenna, M., Chen, C., Qiao, C., Pu, D., Hu, X., Wang, D.Z., Li, J., and Xiao, X. (2009). A myocardium tropic adeno-associated virus (AAV) evolved by DNA shuffling and in vivo selection. Proc. Natl. Acad. Sci. USA *106*, 3946–3951.
42. Zincarelli, C., Soltys, S., Rengo, G., and Rabinowitz, J.E. (2008). Analysis of AAV serotypes 1-9 mediated gene expression and tropism in mice after systemic injection. Mol. Ther. *16*, 1073–1080.
43. Grompe, M., and Strom, S. (2013). Mice with human livers. Gastroenterology *145*, 1209–1214.
44. Arad, U. (1998). Modified Hirt procedure for rapid purification of extrachromosomal DNA from mammalian cells. Biotechniques *24*, 760–762.

OMTM, Volume ■ ■ ■

Supplemental Information

Directed Evolution of AAV Serotype 5 for Increased Hepatocyte Transduction and Retained Low Humoral Seroreactivity

Randolph Qian, Bin Xiao, Juan Li, and Xiao Xiao

Supplemental Figures

>MV1

MSFVDHPPDWLEEVGEGLREFLGLEAGPPKPKPNQQHQDQARGLVLPGYNYLGPGNGLDRGEP
VNRADEVAREHDISYNEQLEAGDNPYLKYNHADAEFQEKLADDTSFGGNLGKAVFQAKKRVLE
PFGLVEEGAKTAPTGKRIDDHFPKRKKARTEEDSKPSTSSDAEAGPSGSQQLQIPAQPASSLGADT
MSAGGGGPLGDNNQGADGVGNASGDWHCDSTWMGDRVVTKSTRTWVLPSYNNHQYREIKSG
SVDRSNANAYFGYSTPWGYFDFNRFHSHWSPRDWQRLINNYWGFRPRSLRVKIFNIQVKEVTVQ
DSTTTIANNLTSTVQVFTDDDYQLPYVVGNGTEGCLPAFPPQVFTLPQYGYATLNRDNTENPTER
SSFFCLEYFPSKMLRTGNNFEFTYNFEEVPFHSSLAPSQNLFKLANPLVDQYLYRFVSTNNTGGV
QFNKNLAGRYANTYKNWFPGPMGRTQGWNLGSGVNRASVSAFATTNRMELEGASYQVPPQPN
GMTNNLQGSNTYALENTMIFNSQPANPGTTATYLEGNMLITSESETQPVNRVAYNVGGQMATN
NQSSTTAPATGTYNLQEIVPGSVWMERDVYLQGPIWAKIPETGAHFHPSPAMGGFGLKHPPPMM
LIKNTPVPGNITSFSDVPVSSFITQYSTGQVTVEMEWELKKENSKRWNPEIQYTNNYNDPQFVDF
APDSTGEYRSTRPIGTRYLTRPL

>MV18

MSFVDHPPDWLEEVGEGLREFLGLEAGPPKPKPNQQHQDQARGLVLPGYNYLGPGNGLDRGEP
VNRADEVAREHDISYNEQLEAGDNPYLKYNHADAEFQEKLADDTSFGGNLGKAVFQAKKRVLE
PFGLVEEGAKTAPTGKRIDDHFPKRKKARTEEDSKPSTSSDAEAGPSGSQQLQIPAQPASSLGADT
MSAGGGGPLGDNNQGADGVGNASGDWHCDSTWMGDRVVTKSTRTWVLPSYNNHQYREIKSG
SVDRSNANAYFGYSTPWGYFDFNRFHSHWSPRDWQRLINNYWGFRPRSLRVKIFNIQVKEVTVQ
DSTTTIANNLTSTVQVFTDDDYQLPYVVGNGTEGCLPAFPPQVFTLPQYGYATLNRDNTENPTER
SSFFCLEYFPSKMLRTGNNFEFTYNFEEVPFHSSFAPSQNLFKLANPLVDQYLYRFVSTNNTGGV
QFNKNLAGRYANTYKNWFPGPMGRTQGWNLGSGVNRASVSAFATTNRMELEGASYQVPPQPN
GMTNNLQGSNTYALENTMIFNSQPANPGTTATYLEGNMLITSESETQPVNRVAYNVGGQMATN
NQSSTTAPATGTYNLQEIVPGSVWMERDVYLQGPIWAKIPETGAHFHPSPAMGGFGLKHPPPMM
LIKNTPVPGNITSFSDVPVSSFITQYSTGQVTVEMEWELKKENSKRWNPEIQYTNNYNDPQFVDF
APDSTGEYRTTRPIGTRYLTRPL

>MV20

MSFVDHPPDWLEEVGEGLREFLGLEAGPPKPKPNQQHQDQARGLVLPGYNYLGPGNGLDRGEP
VNRADEVAREHDISYNEQLEAGDNPYLKYNHADAEFQEKLADDTSFGGNLGKAVFQAKKRVLE
PFGLVEEGAKTAPTGKRIDDHFPKRKKARTEEDSKPSTSSDAEAGPSGSQQLQIPAQPASSLGADT
MSAGGGGSLGDNNQGADGVGNASGDWHCDSTWMGDRVVTKSTRTWVLPSYNNHQYREIKSG
SVDGSNANAYFGYSTPWGYFDFNRFHSHWSPRDWQRLINNYWGFRPRSLRVKIFNIQVKEVTV
QDSTTTIANNLTSTVQVFTDDDYQLPYVVGNGTEGCLPAFPPQVFTLPQYGYATLNRDNTENPTE
RSSFFCLEYFPSKMLRTGNNFEFTYNFEEVPFHSSFAPSQNLFKLANPLVDQYLYRFVSTNNTGG
VQFNKNLAGRYANTYKNWFPGPIGRTQGWNLGSGVNRASVSAFATTNRMELEGASYQVPPQPN
GMTNNLQGSNTYALENTMIFNSQPANPGTTATYLEGNMLITSESETQPVNRVAYNVGGQMATN
NQSSTTTPATGTYNLQEIVPGSVWMERDVYLQGPIWAKIPETGAHFHPSPAMGGFGLKHPPPMM
LIKNTPVPGNITSFSDVPVSSFITQYSTGQVTVEMEWELKKENSKRWNPEIQYTNNYNDPQFVDF
APDSTGEYRSTRPIGTRYLTRPL

>MV50

MSFVDHPPDWLEEVGEGLREFLGLEAGPPKPKPNQQHQDQARGLVLPGYNYLGPGNGLDRGEP
VNRADEVAREHDISYNEQLEAGDNPYLKYNHADAEFQEKLADDTSFGGNLGKAVFQAKKRVLE
PFGLVEEGAKTAPTGKRIDDHFPKRKKARTEEDSKPSTSSDAEAGPSGSQQLQIPAQPASSLGADT
MSAGGGGPLGDNNQGADGVGNASGDWHCDSTWMGDRVVTKSTRTWVLPSYNNHQYREIKSG
SVDRSNANAYFGYSTPWGYFDFNRFHSHWSPRDWQRLINNYWGFRPRSLRVKIFNIQVKEVTVQ
DSTTTIANNLTSTVQVFTDDDYQLPYVVGNGTEGCLPAFPPQVFTLPQYGYATLNRDNTENPTER
SSFFCLEYFPSKMLRTGNNFEFTYNFEEVPFHSSFAPSQNLFKLANPLVDQYLYRFVSTNNTGGV
QFNKNLAGRYANTYKNWFPGPMGRTQGWNLGSGVNRASVSAFATTNRMELEGASYQVPPQPN
GMTNNLQGSNTYALENTMIFNSQPANPGTTATYLEGNMLITSESETQPVNRVAYNVGGQMATN
NQSSTTAPATGTYNLQEIVPGSVWMERDVYLQGPIWAKIPETGAHFHPSPAMGGFGLKHPPPMM
LIKNTPVPGNITSFSDVPVSSFITQYSTGQVTVEMEWELKKENSKRWNPEIQYTNNYNDPQFVDF
APDGTGEYRSTRPIGTRYLTRPL

>MV53

MSFVDHPPDWLEEVGEGLREFLGLEAGPPKPKPNQQHQDQARGLVLPGYNYLGPGNGLDRGEP
VNRADEVAREHDISYNEQLEAGDNPYLKYNHADAEFQEKLADDTSFGGNLGKAVFQAKKRVLE
PFGLVEEGAKTAPTGKRIDDHFPKRKKARTEEDSKPSTSSDAEAGPSGSQQLRIPAQPASSLGADT
MSAGGGGPLGDNNQGADGVGNASGDWHCDSTWMGDRVVTKSTRTWVLPSYNNHQYREIKSG
SVDRSNANAYFGYSTPWGYFDFNRFHSHWSPRDWQRLINNYWGFRPRSLRVKIFNIQVKEVTVQ
DSTTTIANNLTSTVQVFTDDDYQLPYVVGNGTEGCLPAFPPQVFTLPQYGYATLNRDNTENPTER
SSFFCLEYFPSKMLRTGNNFEFTYNFEEVPFHSSFAPSQNLFKLANPLVDQYLYRFVSTNNTGGV
QFNKNLAGRYANTYKNWFPGPMGRTQGWNLGSGVNRASVSAFATTNRMELEGASYQVPPQPN
GMTNNLQGSNTYALENTMIFNSQPANPGTTATYLEGNMLITSESETQPVNRVAYNVGGQMATN
NQSSTTAPATGTYNLQEIVPGSVWMERDVYLQGPIWAKIPETGAHFHPSPAMGGFGLKHPPPMM
LIKNTPVPGNITSFSDVPVSSFITQYSTGQVTVEMEWELKKENSKRWNPEIQYTNNYNDPQFVDF
APDSTGEYRTTRPIGTRYLTRPL

Figure S1: Amino Acid Sequence for Best Performing AAV5 Variants

Amino acid sequences for best performing variants. The mutations that differ from AAV5 are highlighted in red.

```
AAV5     1 MSFVDHPPDWLEEVGEGLREFLGLEAGPPKPKPNQQHQDQARGLVLPGYNYLGPGNGLDR
MV1      1 MSFVDHPPDWLEEVGEGLREFLGLEAGPPKPKPNQQHQDQARGLVLPGYNYLGPGNGLDR
MV18     1 MSFVDHPPDWLEEVGEGLREFLGLEAGPPKPKPNQQHQDQARGLVLPGYNYLGPGNGLDR
MV20     1 MSFVDHPPDWLEEVGEGLREFLGLEAGPPKPKPNQQHQDQARGLVLPGYNYLGPGNGLDR
MV50     1 MSFVDHPPDWLEEVGEGLREFLGLEAGPPKPKPNQQHQDQARGLVLPGYNYLGPGNGLDR
MV53     1 MSFVDHPPDWLEEVGEGLREFLGLEAGPPKPKPNQQHQDQARGLVLPGYNYLGPGNGLDR

AAV5    61 GEPVNRADEVAREHDISYNEQLEAGDNPYLKYNHADAEFQEKLADDTSFGGNLGKAVFQA
MV1     61 GEPVNRADEVAREHDISYNEQLEAGDNPYLKYNHADAEFQEKLADDTSFGGNLGKAVFQA
MV18    61 GEPVNRADEVAREHDISYNEQLEAGDNPYLKYNHADAEFQEKLADDTSFGGNLGKAVFQA
MV20    61 GEPVNRADEVAREHDISYNEQLEAGDNPYLKYNHADAEFQEKLADDTSFGGNLGKAVFQA
MV50    61 GEPVNRADEVAREHDISYNEQLEAGDNPYLKYNHADAEFQEKLADDTSFGGNLGKAVFQA
MV53    61 GEPVNRADEVAREHDISYNEQLEAGDNPYLKYNHADAEFQEKLADDTSFGGNLGKAVFQA

AAV5   121 KKRVLEPFGLVEEGAKTAPTGKRIDDHFPKRKKARTEEDSKPSTSSDAEAGPSGSQQLQI
MV1    121 KKRVLEPFGLVEEGAKTAPTGKRIDDHFPKRKKARTEEDSKPSTSSDAEAGPSGSQQLQI
MV18   121 KKRVLEPFGLVEEGAKTAPTGKRIDDHFPKRKKARTEEDSKPSTSSDAEAGPSGSQQLQI
MV20   121 KKRVLEPFGLVEEGAKTAPTGKRIDDHFPKRKKARTEEDSKPSTSSDAEAGPSGSQQLQI
MV50   121 KKRVLEPFGLVEEGAKTAPTGKRIDDHFPKRKKARTEEDSKPSTSSDAEAGPSGSQQLQI
MV53   121 KKRVLEPFGLVEEGAKTAPTGKRIDDHFPKRKKARTEEDSKPSTSSDAEAGPSGSQQLRI

AAV5   181 PAQPASSLGADTMSAGGGGPLGDNNQGADGVGNASGDWHCDSTWMGDRVVTKSTRTWVLP
MV1    181 PAQPASSLGADTMSAGGGGPLGDNNQGADGVGNASGDWHCDSTWMGDRVVTKSTRTWVLP
MV18   181 PAQPASSLGADTMSAGGGGPLGDNNQGADGVGNASGDWHCDSTWMGDRVVTKSTRTWVLP
MV20   181 PAQPASSLGADTMSAGGGGSLGDNNQGADGVGNASGDWHCDSTWMGDRVVTKSTRTWVLP
MV50   181 PAQPASSLGADTMSAGGGGPLGDNNQGADGVGNASGDWHCDSTWMGDRVVTKSTRTWVLP
MV53   181 PAQPASSLGADTMSAGGGGPLGDNNQGADGVGNASGDWHCDSTWMGDRVVTKSTRTWVLP

AAV5   241 SYNNHQYREIKSGSVDGSNANAYFGYSTPWGYFDFNRFHSHWSPRDWQRLINNYWGFRPR
MV1    241 SYNNHQYREIKSGSVDRSNANAYFGYSTPWGYFDFNRFHSHWSPRDWQRLINNYWGFRPR
MV18   241 SYNNHQYREIKSGSVDRSNANAYFGYSTPWGYFDFNRFHSHWSPRDWQRLINNYWGFRPR
MV20   241 SYNNHQYREIKSGSVDGSNANAYFGYSTPWGYFDFNRFHSHWSPRDWQRLINNYWGFRPR
MV50   241 SYNNHQYREIKSGSVDRSNANAYFGYSTPWGYFDFNRFHSHWSPRDWQRLINNYWGFRPR
MV53   241 SYNNHQYREIKSGSVDRSNANAYFGYSTPWGYFDFNRFHSHWSPRDWQRLINNYWGFRPR

AAV5   301 SLRVKIFNIQVKEVTVQDSTTTIANNLTSTVQVFTDDDYQLPYVVGNGTEGCLPAFPPQV
MV1    301 SLRVKIFNIQVKEVTVQDSTTTIANNLTSTVQVFTDDDYQLPYVVGNGTEGCLPAFPPQV
MV18   301 SLRVKIFNIQVKEVTVQDSTTTIANNLTSTVQVFTDDDYQLPYVVGNGTEGCLPAFPPQV
MV20   301 SLRVKIFNIQVKEVTVQDSTTTIANNLTSTVQVFTDDDYQLPYVVGNGTEGCLPAFPPQV
MV50   301 SLRVKIFNIQVKEVTVQDSTTTIANNLTSTVQVFTDDDYQLPYVVGNGTEGCLPAFPPQV
MV53   301 SLRVKIFNIQVKEVTVQDSTTTIANNLTSTVQVFTDDDYQLPYVVGNGTEGCLPAFPPQV

AAV5   361 FTLPQYGYATLNRDNTENPTERSSFFCLEYFPSKMLRTGNNFEFTYNFEEVPFHSSFAPS
MV1    361 FTLPQYGYATLNRDNTENPTERSSFFCLEYFPSKMLRTGNNFEFTYNFEEVPFHSSLAPS
MV18   361 FTLPQYGYATLNRDNTENPTERSSFFCLEYFPSKMLRTGNNFEFTYNFEEVPFHSSFAPS
MV20   361 FTLPQYGYATLNRDNTENPTERSSFFCLEYFPSKMLRTGNNFEFTYNFEEVPFHSSFAPS
MV50   361 FTLPQYGYATLNRDNTENPTERSSFFCLEYFPSKMLRTGNNFEFTYNFEEVPFHSSFAPS
MV53   361 FTLPQYGYATLNRDNTENPTERSSFFCLEYFPSKMLRTGNNFEFTYNFEEVPFHSSFAPS

AAV5   421 QNLFKLANPLVDQYLYRFVSTNNTGGVQFNKNLAGRYANTYKNWFPGPMGRTQGWNLGSG
```

```
MV1   421 QNLFKLANPLVDQYLYRFVSTNNTGGVQFNKNLAGRYANTYKNWFPGPMGRTQGWNLGSG
MV18  421 QNLFKLANPLVDQYLYRFVSTNNTGGVQFNKNLAGRYANTYKNWFPGPMGRTQGWNLGSG
MV20  421 QNLFKLANPLVDQYLYRFVSTNNTGGVQFNKNLAGRYANTYKNWFPGPIGRTQGWNLGSG
MV50  421 QNLFKLANPLVDQYLYRFVSTNNTGGVQFNKNLAGRYANTYKNWFPGPMGRTQGWNLGSG
MV53  421 QNLFKLANPLVDQYLYRFVSTNNTGGVQFNKNLAGRYANTYKNWFPGPMGRTQGWNLGSG

AAV5  481 VNRASVSAFATTNRMELEGASYQVPPQPNGMTNNLQGSNTYALENTMIFNSQPANPGTTA
MV1   481 VNRASVSAFATTNRMELEGASYQVPPQPNGMTNNLQGSNTYALENTMIFNSQPANPGTTA
MV18  481 VNRASVSAFATTNRMELEGASYQVPPQPNGMTNNLQGSNTYALENTMIFNSQPANPGTTA
MV20  481 VNRASVSAFATTNRMELEGASYQVPPQPNGMTNNLQGSNTYALENTMIFNSQPANPGTTA
MV50  481 VNRASVSAFATTNRMELEGASYQVPPQPNGMTNNLQGSNTYALENTMIFNSQPANPGTTA
MV53  481 VNRASVSAFATTNRMELEGASYQVPPQPNGMTNNLQGSNTYALENTMIFNSQPANPGTTA

AAV5  541 TYLEGNMLITSESETQPVNRVAYNVGGQMATNNQSSTTAPATGTYNLQEIVPGSVWMERD
MV1   541 TYLEGNMLITSESETQPVNRVAYNVGGQMATNNQSSTTAPATGTYNLQEIVPGSVWMERD
MV18  541 TYLEGNMLITSESETQPVNRVAYNVGGQMATNNQSSTTAPATGTYNLQEIVPGSVWMERD
MV20  541 TYLEGNMLITSESETQPVNRVAYNVGGQMATNNQSSTTTPATGTYNLQEIVPGSVWMERD
MV50  541 TYLEGNMLITSESETQPVNRVAYNVGGQMATNNQSSTTAPATGTYNLQEIVPGSVWMERD
MV53  541 TYLEGNMLITSESETQPVNRVAYNVGGQMATNNQSSTTAPATGTYNLQEIVPGSVWMERD

AAV5  601 VYLQGPIWAKIPETGAHFHPSPAMGGFGLKHPPPMMLIKNTPVPGNITSFSDVPVSSFIT
MV1   601 VYLQGPIWAKIPETGAHFHPSPAMGGFGLKHPPPMMLIKNTPVPGNITSFSDVPVSSFIT
MV18  601 VYLQGPIWAKIPETGAHFHPSPAMGGFGLKHPPPMMLIKNTPVPGNITSFSDVPVSSFIT
MV20  601 VYLQGPIWAKIPETGAHFHPSPAMGGFGLKHPPPMMLIKNTPVPGNITSFSDVPVSSFIT
MV50  601 VYLQGPIWAKIPETGAHFHPSPAMGGFGLKHPPPMMLIKNTPVPGNITSFSDVPVSSFIT
MV53  601 VYLQGPIWAKIPETGAHFHPSPAMGGFGLKHPPPMMLIKNTPVPGNITSFSDVPVSSFIT

AAV5  661 QYSTGQVTVEMEWELKKENSKRWNPEIQYTNNYNDPQFVDFAPDSTGEYRTTRPIGTRYL
MV1   661 QYSTGQVTVEMEWELKKENSKRWNPEIQYTNNYNDPQFVDFAPDSTGEYRTTRPIGTRYL
MV18  661 QYSTGQVTVEMEWELKKENSKRWNPEIQYTNNYNDPQFVDFAPDSTGEYRTTRPIGTRYL
MV20  661 QYSTGQVTVEMEWELKKENSKRWNPEIQYTNNYNDPQFVDFAPDSTGEYRTTRPIGTRYL
MV50  661 QYSTGQVTVEMEWELKKENSKRWNPEIQYTNNYNDPQFVDFAPDGTGEYRTTRPIGTRYL
MV53  661 QYSTGQVTVEMEWELKKENSKRWNPEIQYTNNYNDPQFVDFAPDSTGEYRTTRPIGTRYL

AAV5  721 TRPL
MV1   721 TRPL
MV18  721 TRPL
MV20  721 TRPL
MV50  721 TRPL
MV53  721 TRPL
```

Figure S2: Amino Acid Sequence Alignment of AAV5 and MV Mutants

Alignment of the amino acid sequences for each of the MV mutants and AAV5. The mutations that differ from AAV5 are highlighted in black with white font.

```
AAV1            MAADGYLPDWLEDNLSEGIREWWDLKPGAPKPKANQQKQDDGRGLVLPGYKYLGPFNGLD
AAV6            MAADGYLPDWLEDNLSEGIREWWDLKPGAPKPKANQQKQDDGRGLVLPGYKYLGPFNGLD
AAV2            MAADGYLPDWLEDTLSEGIRQWWKLKPGPPPPKPAERHKDDSRGLVLPGYKYLGPFNGLD
AAV3            MAADGYLPDWLEDNLSEGIREWWALKPGVPQPKANQQHQDNRRGLVLPGYKYLGPGNGLD
AAV7            MAADGYLPDWLEDNLSEGIREWWDLKPGAPKPKANQQKQDNGRGLVLPGYKYLGPFNGLD
AAV8            MAADGYLPDWLEDNLSEGIREWWALKPGAPKPKANQQKQDDGRGLVLPGYKYLGPFNGLD
AAV9            MAADGYLPDWLEDNLSEGIREWWALKPGAPQPKANQQHQDNARGLVLPGYKYLGPGNGLD
AAV4            -MTDGYLPDWLEDNLSEGVREWWALQPGAPKPKANQQHQDNARGLVLPGYKYLGPGNGLD
MV              MSFVDHPPDWLEEVG-EGLREFLGLEAGPPKPKPNQQHQDQARGLVLPGYNYLGPGNGLD
                    .: *****:   **:*::  *:.* * **. ::::*: ********:**** ****

AAV1            KGEPVNAADAAALEHDKAYDQQLKAGDNPYLRYNHADAEFQERLQEDTSFGGNLGRAVFQ
AAV6            KGEPVNAADAAALEHDKAYDQQLKAGDNPYLRYNHADAEFQERLQEDTSFGGNLGRAVFQ
AAV2            KGEPVNEADAAALEHDKAYDRQLDSGDNPYLKYNHADAEFQERLKEDTSFGGNLGRAVFQ
AAV3            KGEPVNEADAAALEHDKAYDQQLKAGDNPYLKYNHADAEFQERLQEDTSFGGNLGRAVFQ
AAV7            KGEPVNAADAAALEHDKAYDQQLKAGDNPYLRYNHADAEFQERLQEDTSFGGNLGRAVFQ
AAV8            KGEPVNAADAAALEHDKAYDQQLQAGDNPYLRYNHADAEFQERLQEDTSFGGNLGRAVFQ
AAV9            KGEPVNAADAAALEHDKAYDQQLKAGDNPYLKYNHADAEFQERLKEDTSFGGNLGRAVFQ
AAV4            KGEPVNAADAAALEHDKAYDQQLKAGDNPYLKYNHADAEFQQRLQGDTSFGGNLGRAVFQ
MV              RGEPVNRADEVAREHDISYNEQLEAGDNPYLKYNHADAEFQEKLADDTSFGGNLGKAVFQ
                :***** ** .* *** :*:.**.:******:*********::*  *********:****

AAV1            AKKRVLEPLGLVEEGAKTAPGKKRPVEQSPQE-PDSSSGIGKTGQQPAKKRLNFGQTGDS
AAV6            AKKRVLEPFGLVEEGAKTAPGKKRPVEQSPQE-PDSSSGIGKTGQQPAKKRLNFGQTGDS
AAV2            AKKRVLEPLGLVEEPVKTAPGKKRPVEHSPVE-PDSSSGTGKAGQQPARKRLNFGQTGDA
AAV3            AKKRILEPLGLVEEAAKTAPGKKRPVDQSPQE-PDSSSGVGKSGKQPARKRLNFGQTGDS
AAV7            AKKRVLEPLGLVEEGAKTAPAKKRPVEPSPQRSPDSSTGIGKKGQQPARKRLNFGQTGDS
AAV8            AKKRVLEPLGLVEEGAKTAPGKKRPVEPSPQRSPDSSTGIGKKGQQPARKRLNFGQTGDS
AAV9            AKKRLLEPLGLVEEAAKTAPGKKRPVEQSPQE-PDSSAGIGKSGAQPAKKRLNFGQTGDT
AAV4            AKKRVLEPLGLVEQAGETAPGKKRPLIESPQQ-PDSSTGIGKKGKQPAKKKLVFEDETGA
MV              AKKRVLEPFGLVEEGAKTAPTGKRIDDHFPKR----------KKARTEEDSKPSTSSDAE
                ****:***:****:  :***  **     * .             :. ..     .

AAV1            ESVPDPQPLGEPPATPAAVGPTTMASGGGAPMADNNEGADGVGNASGNWHCDSTWLGDRV
AAV6            ESVPDPQPLGEPPATPAAVGPTTMASGGGAPMADNNEGADGVGNASGNWHCDSTWLGDRV
AAV2            DSVPDPQPLGQPPAAPSGLGTNTMATGSGAPMADNNEGADGVGNSSGNWHCDSTWMGDRV
AAV3            ESVPDPQPLGEPPAAPTSLGSNTMASGGGAPMADNNEGADGVGNSSGNWHCDSQWLGDRV
AAV7            ESVPDPQPLGEPPAAPSSVGSGTVAAGGGAPMADNNEGADGVGNASGNWHCDSTWLGDRV
AAV8            ESVPDPQPLGEPPAAPSGVGPNTMAAGGGAPMADNNEGADGVGSSSGNWHCDSTWLGDRV
AAV9            ESVPDPQPIGEPPAAPSGVGSLTMASGGGAPVADNNEGADGVGSSSGNWHCDSQWLGDRV
AAV4            GDGPP-----EGSTSGAMSDDSEMRAAAGGAAVEGGQGADGVGNASGDWHCDSTWSEGHV
MV              AGPSGSQQLRIPAQPASSLGADTMSAGGGGPLGDNNQGADGVGNASGDWHCDSTWMGDRV
                 . .        . . :  .   : :..*..  :..:******.:**:***** *  .:*

AAV1            ITTSTRTWALPTYNNHLYK---QIS-SASTGASNDNHYFGYSTPWGYFDFNRFHCHFSPR
AAV6            ITTSTRTWALPTYNNHLYK---QIS-SASTGASNDNHYFGYSTPWGYFDFNRFHCHFSPR
AAV2            ITTSTRTWALPTYNNHLYK---QIS-SQSG-ASNDNHYFGYSTPWGYFDFNRFHCHFSPR
AAV3            ITTSTRTWALPTYNNHLYK---QIS-SQSG-ASNDNHYFGYSTPWGYFDFNRFHCHFSPR
AAV7            ITTSTRTWALPTYNNHLYK---QISSETAG-STNDNTYFGYSTPWGYFDFNRFHCHFSPR
AAV8            ITTSTRTWALPTYNNHLYK---QISNGTSGGATNDNTYFGYSTPWGYFDFNRFHCHFSPR
AAV9            ITTSTRTWALPTYNNHLYK---QISNSTSGGSSNDNAYFGYSTPWGYFDFNRFHCHFSPR
AAV4            TTTSTRTWVLPTYNNHLYKRLG--------ESLQSNTYNGFSTPWGYFDFNRFHCHFSPR
MV              VTKSTRTWVLPSYNNHQYR----EIKSGSVDRSNANAYFGYSTPWGYFDFNRFHSHWSPR
                 *.*****.**:**** *:              : * * *:*************.*:***

AAV1            DWQRLINNNWGFRPKRLNFKLFNIQVKEVTTNDGVTTIANNLTSTVQVFSDSEYQLPYVL
AAV6            DWQRLINNNWGFRPKRLNFKLFNIQVKEVTTNDGVTTIANNLTSTVQVFSDSEYQLPYVL
AAV2            DWQRLINNNWGFRPKRLNFKLFNIQVKEVTQNDGTTTIANNLTSTVQVFTDSEYQLPYVL
AAV3            DWQRLINNNWGFRPKKLSFKLFNIQVKEVTQNDGTTTIANNLTSTVQVFTDSEYQLPYVL
AAV7            DWQRLINNNWGFRPKKLRFKLFNIQVKEVTTNDGVTTIANNLTSTIQVFSDSEYQLPYVL
AAV8            DWQRLINNNWGFRPKRLSFKLFNIQVKEVTQNEGTKTIANNLTSTIQVFTDSEYQLPYVL
AAV9            DWQRLINNNWGFRPKRLNFKLFNIQVKEVTDNNGVKTIANNLTSTVQVFTDSDYQLPYVL
AAV4            DWQRLINNNWGMRPKAMRVKIFNIQVKEVTTSNGETTVANNLTSTVQIFADSSYELPYVM
```

```
MV              DWQRLINNYWGFRPRSLRVKIFNIQVKEVTVQDSTTTIANNLTSTVQVFTDDDYQLPYVV
                ******** **:**: : .*:********* .:. .*:*******:*:*:*..*:****:

AAV1            GSAHQGCLPPFPADVFMIPQYGYLTLNNG---SQAVGRSSFYCLEYFPSQMLRTGNNFTF
AAV6            GSAHQGCLPPFPADVFMIPQYGYLTLNNG---SQAVGRSSFYCLEYFPSQMLRTGNNFTF
AAV2            GSAHQGCLPPFPADVFMVPQYGYLTLNNG---SQAVGRSSFYCLEYFPSQMLRTGNNFTF
AAV3            GSAHQGCLPPFPADVFMVPQYGYLTLNNG---SQAVGRSSFYCLEYFPSQMLRTGNNFQF
AAV7            GSAHQGCLPPFPADVFMIPQYGYLTLNNG---SQSVGRSSFYCLEYFPSQMLRTGNNFEF
AAV8            GSAHQGCLPPFPADVFMIPQYGYLTLNNG---SQAVGRSSFYCLEYFPSQMLRTGNNFQF
AAV9            GSAHEGCLPPFPADVFMIPQYGYLTLNDG---SQAVGRSSFYCLEYFPSQMLRTGNNFQF
AAV4            DAGQEGSLPPFPNDVFMVPQYGYCGLVTGNTSQQQTDRNAFYCLEYFPSQMLRTGNNFEI
MV              GNGTEGCLPAFPPQVFTLPQYGYATLNRDN-TENPTERSSFFCLEYFPSKMLRTGNNFEF
                . . :*.**.** :** :*****  *  .   .: . *.:*:*******:******** :

AAV1            SYTFEEVPFHSSYAHSQSLDRLMNPLIDQYLYYLNRTQ-NQSGSAQNKDLLFSRGSPAGM
AAV6            SYTFEDVPFHSSYAHSQSLDRLMNPLIDQYLYYLNRTQ-NQSGSAQNKDLLFSRGSPAGM
AAV2            SYTFEDVPFHSSYAHSQSLDRLMNPLIDQYLYYLSRTN-TPSGTTTQSRLQFSQAGASDI
AAV3            SYTFEDVPFHSSYAHSQSLDRLMNPLIDQYLYYLNRTQGTTSGTTNQSRLLFSQAGPQSM
AAV7            SYSFEDVPFHSSYAHSQSLDRLMNPLIDQYLYYLARTQSNPGGTAGNRELQFYQGGPSTM
AAV8            TYTFEDVPFHSSYAHSQSLDRLMNPLIDQYLYYLSRTQTTGG-TANTQTLGFSQGGPNTM
AAV9            SYEFENVPFHSSYAHSQSLDRLMNPLIDQYLYYLSKTINGSG--QNQQTLKFSVAGPSNM
AAV4            TYSFEKVPFHSMYAHSQSLDRLMNPLIDQYLWGLQSTTTGTTLNAGTATTNFTKLRPTNF
MV              TYNFEEVPFHSSLAPSQNLFKLANPLVDQYLYRFVSTN-------NTGGVQFNKNLAGRY
                :* **.*****  * **.* :* ***:****: :  *              *    .

AAV1            SVQPKNWLPGPCYRQQRVSKTKTD-----NNNSNFTWTGASKYNLNGRESIINPGTAMAS
AAV6            SVQPKNWLPGPCYRQQRVSKTKTD-----NNNSNFTWTGASKYNLNGRESIINPGTAMAS
AAV2            RDQSRNWLPGPCYRQQRVSKTSAD-----NNNSEYSWTGATKYHLNGRDSLVN--PAMAS
AAV3            SLQARNWLPGPCYRQQRLSKTAND-----NNNSNFPWTAASKYHLNGRDSLVNPGPAMAS
AAV7            AEQAKNWLPGPCFRQQRVSKTLDQ-----NNNSNFAWTGATKYHLNGRNSLVNPGVAMAT
AAV8            ANQAKNWLPGPCYRQQRVSTTTGQ-----NNNSNFAWTAGTKYHLNGRNSLANPGIAMAT
AAV9            AVQGRNYIPGPSYRQQRVSTTVTQ-----NNNSEFAWPGASSWALNGRNSLMNPGPAMAS
AAV4            SNFKKNWLPGPSIKQQGFSKTANQNYKIPATGSDSLIKYETHSTLDGRWSALTPGPPMAT
MV              ANTYKNWFPGPMGRTQGWNLGSGVN-----RASVSAFATTNRMELEGASYQVPPQPNGMT
                    :*::***  : *  .             *       .   *:*            :

AAV1            HKDDEDKFFPMSGVMIFGKESAGA---SNTALDNVMITDEEEIKATNPVATERFGTVAVN
AAV6            HKDDKDKFFPMSGVMIFGKESAGA---SNTALDNVMITDEEEIKATNPVATERFGTVAVN
AAV2            HKDDEEKFFPQSGVLIFGKQGSEK---TNVNIEKVMITDEEEIGTTNPVATEQYGSVSTN
AAV3            HKDDEEKFFPMHGNLIFGKEGTTA---SNAELDNVMITDEEEIRTTNPVATEQYGTVANN
AAV7            HKDDEDRFFPSSGVLIFG-KTGAT---NKTTLENVLMTNEEEIRPTNPVATEEYGIVSSN
AAV8            HKDDEERFFPSNGILIFGKQNAAR---DNADYSDVMLTSEEEIKTTNPVATEEYGIVADN
AAV9            HKEGEDRFFPLSGSLIFGKQGTGR---DNVDADKVMITNEEEIKTTNPVATESYGQVATN
AAV4            AGPADSKFS-NSQLIFAGPKQNGN---TATVPGTLIFTSEEELAATNATDTDMWGNLPGG
MV              NNLQGSNTYALENTMIFNSQPANPGTTATYLEGNMLITSESETQPVNRVAYNVGGQMATN
                     ..       :: . :              :::*.*.*  ..* .  :  * :. .

AAV1            FQSSSTDPATGDVHAMGALPGMVWQDRDVYLQGPIWAKIPHTDGHFHPSPLMGGFGLKNP
AAV6            LQSSSTDPATGDVHVMGALPGMVWQDRDVYLQGPIWAKIPHTDGHFHPSPLMGGFGLKHP
AAV2            LQRGNRQAATADVNTQGVLPGMVWQDRDVYLQGPIWAKIPHTDGHFHPSPLMGGFGLKHP
AAV3            LQSSNTAPTTRTVNDQGALPGMVWQDRDVYLQGPIWAKIPHTDGHFHPSPLMGGFGLKHP
AAV7            LQAANTAAQTQVVNNQGALPGMVWQNRDVYLQGPIWAKIPHTDGNFHPSPLMGGFGLKHP
AAV8            LQQQNTAPQIGTVNSQGALPGMVWQNRDVYLQGPIWAKIPHTDGNFHPSPLMGGFGLKHP
AAV9            HQSAQAQAQTGWVQNQGILPGMVWQDRDVYLQGPIWAKIPHTDGNFHPSPLMGGFGMKHP
AAV4            DQSNSNLPTVDRLTALGAVPGMVWQNRDIYYQGPIWAKIPHTDGHFHPSPLIGGFGLKHP
MV              NQSSTTTPATGTYNLQEIVPGSVWMERDVYLQGPIWAKIPETGAHFHPSPAMGGFGLKHP
                 *     .          :** ** :**:* *********.*..:***** :****:*:*

AAV1            PPQILIKNTPVPANPPAEFSATKFASFITQYSTGQVSVEIEWELQKENSKRWNPEVQYTS
AAV6            PPQILIKNTPVPANPPAEFSATKFASFITQYSTGQVSVEIEWELQKENSKRWNPEVQYTS
AAV2            PPQILIKNTPVPANPSTTFSAAKFASFITQYSTGQVSVEIEWELQKENSKRWNPEIQYTS
AAV3            PPQIMIKNTPVPANPPTTFSPAKFASFITQYSTGQVSVEIEWELQKENSKRWNPEIQYTS
AAV7            PPQILIKNTPVPANPPEVFTPAKFASFITQYSTGQVSVEIEWELQKENSKRWNPEIQYTS
```

```
AAV8            PPQILIKNTPVPADPPTTFNQSKLNSFITQYSTGQVSVEIEWELQKENSKRWNPEIQYTS
AAV9            PPQILIKNTPVPADPPTAFNKDKLNSFITQYSTGQVSVEIEWELQKENSKRWNPEIQYTS
AAV4            PPQIFIKNTPVPANPATTFSSTPVNSFITQYSTGQVSVQIDWEIQKERSKRWNPEVQFTS
MV              PPMMLIKNTPVPGN-ITSFSDVPVSSFITQYSTGQVTVEMEWELKKENSKRWNPEIQYTN
                ** ::*******.:    *.   . ***********:*:::**::**.*******:*:*.

AAV1            NYAKSANVDFTVDNNGLYTEPRPIGTRYLTRPL
AAV6            NYAKSANVDFTVDNNGLYTEPRPIGTRYLTRPL
AAV2            NYNKSVNRGLTVDTNGVYSEPRPIGTRYLTRNL
AAV3            NYNKSVNVDFTVDTNGVYSEPRPIGTRYLTRNL
AAV7            NFEKQTGVDFAVDSQGVYSEPRPIGTRYLTRNL
AAV8            NYYKSTSVDFAVNTEGVYSEPRPIGTRYLTRNL
AAV9            NYYKSNNVEFAVNTEGVYSEPRPIGTRYLTRNL
AAV4            NYGQQNSLLWAPDAAGKYTEPRAIGTRYLTHHL
MV              NYNDPQFVDFAPDGTGEYRSTRPIGTRYLTRPL
                *: .      : :  * * ..*.*******: *
```

Figure S3: Alignment of All Mutations found in MV Mutants with Other AAV Serotypes

Alignment of AAV serotypes 1 through 9 against MV which contains all of the mutations found in the MV mutants pooled into one sequence. Mutations are highlighted in red.

A

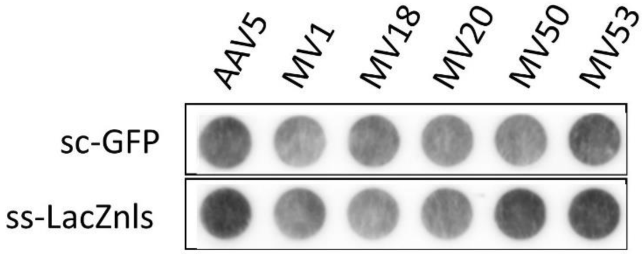

B

AAV5 and MV Mutant Viral Titer

Virus	sc-GFP (vg/ml)	ss-LacZ (vg/ml)
AAV5	2.10E+12	3.78E+12
MV1	1.35E+12	1.46E+12
MV18	1.50E+12	1.96E+12
MV20	1.40E+12	2.36E+12
MV50	1.45E+12	3.54E+12
MV53	2.30E+12	3.76E+12

Figure S4: Production Yield of AAV5 and MV Mutants

(A) DNA dotblot for the yield of each mutant and AAV5 for two versions of the virus, sc-GFP and ss-LacZnls. (B) Production titers for each capsid and each transgene version.

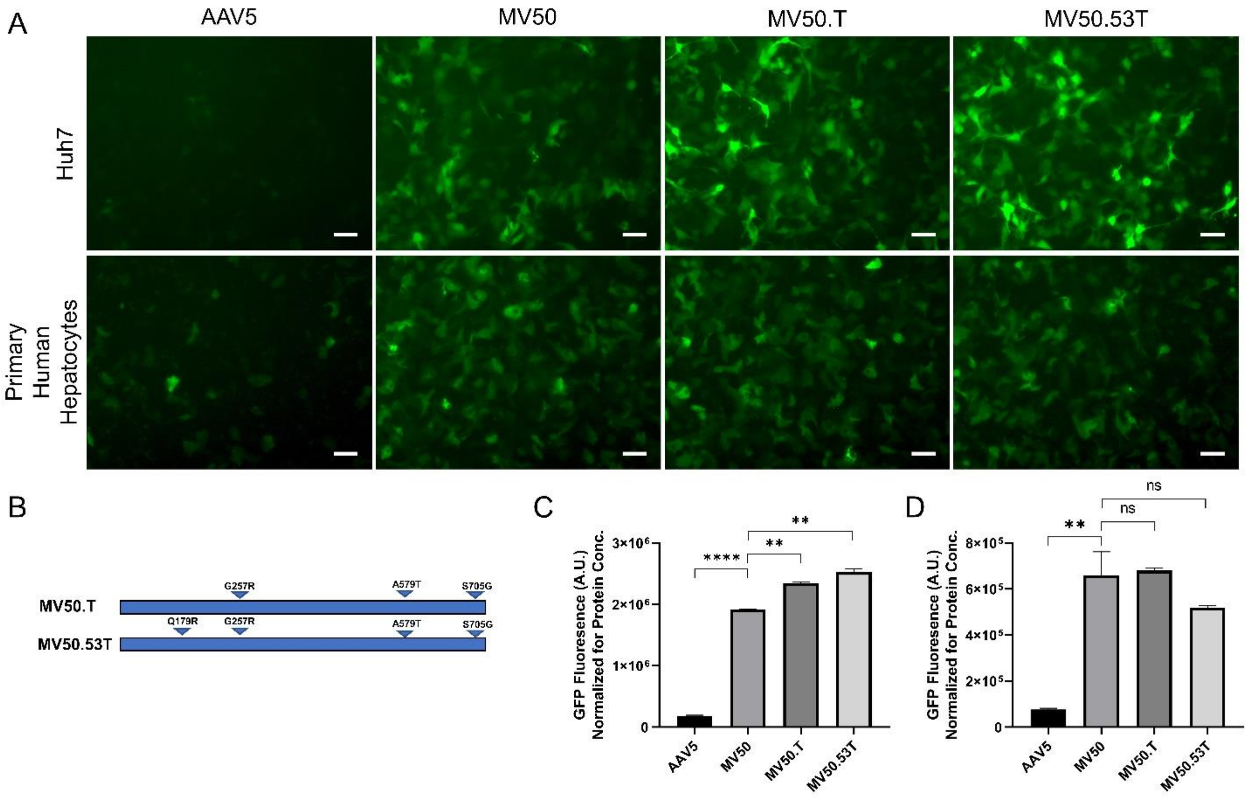

Figure S5. Combination of Mutations from multiple AAV5 Variants

(A) Fluorescent imaging of Huh7 cells and primary human hepatocytes(TRL HUM4020) transduced with self-complementary GFP vectors with AAV5, MV50, MV50.T, and MV50.53T. Huh7 cells were transduced at a MOI of 1e5 vg/cell and human hepatocytes were transduced at a MOI of 5e5vg/cell. Images were taken 96 hours after infection. Expression of GFP (green) indicates transduced cells. 10x magnification. Scale Bar: 100um. (B) Depiction of the combined mutations that are in MV50.T and MV50.53T. Location of amino acid mutations in the AAV5 VP1 amino acid sequence are indicated by triangles. The exact mutation is indicated above the triangle. (C) Comparison of normalized GFP expression in Huh7 cells between AAV5, MV50, MV50.T, and MV50.53T. Data are shown as mean values ± SD. **p<0.01; ****p<0.0001. (D) Comparison of normalized GFP expression in primary human hepatocytes between AAV5, MV50, MV50.T, and MV50.53T. Data are shown as mean values ± SD. **p<0.01.

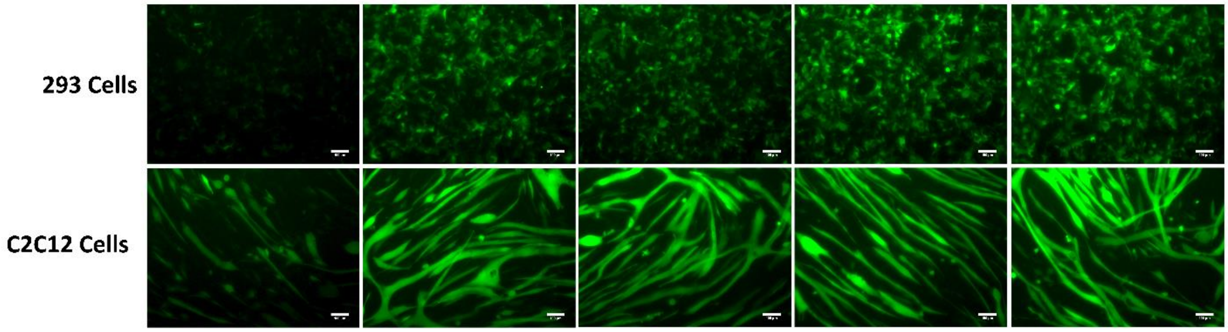

Figure S6. Transduction Efficiency of MV Mutants Compared to AAV5 in 293 and Differentiated C2C12 Cells

(A) Fluorescent imaging of 293 cells and differentiated C2C12 cells transduced with self-complementary GFP vectors packaged by AAV5, MV1, MV18, MV50, and MV53. Both cell lines were transduced at a MOI of 4e5 vg/cell. Images were taken 96 hours after infection. Expression of GFP (green) indicates transduced cells. 10x magnification. Scale Bar: 100um.

Table S1: Replacement of AAV5 Loop1/VRI Amino Acid Sequence with Other AAV Serotype Loop1

AAV Serotype	Loop1 Sequence	Packages DNA?	Infectious on 293?	Infectious in Mice?
1	ISSASTGASNDNHY	No	No	No
2	ISSQSGASNDNHY	No	No	No
4	LGESLQSNTYN	No	No	No
7	ISSETAGSTNDNTY	Yes	No	No
8	ISNGTSGGATNDNTY	No	No	No
9	ISNGTSGGSTNDNTY	No	No	No
Avian	IQGPGGTDPNNKFF	Yes	No	No

Replacement of AAV5 Loop1/VRI amino acid sequence with other AAV serotypes Loop1/VRI sequence. The sequence IKSGSVDGSNANAY was replaced with the sequence indicated in the table using PCR-based cloning. Modified AAV5 capsids were then used to package DNA and tested for their infectivity in 293 cells and mice.

Supplemental Methods

Transduction of 293 Cells by AAV Vectors

Variant AAV5 vectors were used to packaged sc-GFP reporter genes for comparison to wt-AAV5. 293 cells in 12 well plates were infected with AAV5 and mutant self-complementary(sc)-GFP encoding viruses at a MOI of 400,000 viral genomes(vg) per cell and co-infected with 5000 vg/cell of wt-ad5. Ninety-six hours after infection, cells were taken for fluorescent imaging.

Transduction of Differentiated C2C12 Cells by AAV Vectors

Variant AAV5 vectors were used to packaged sc-GFP reporter genes for comparison to wt-AAV5. C2C12 myoblasts were seeded in 12 well plates were cultured with DMEM supplemented with 10% FBS and 1% penicillin/streptomycin (p/s). Upon reaching 70% confluency, culture medium was changed to differentiated medium (DMEM with 2% horse serum and 1% p/s). C2C12 cells were differentiated for five days prior to infection with AAV5 and mutant self-complementary(sc)-GFP encoding viruses at a MOI of 400,000 viral genomes(vg) per cell and co-infected with 5000 vg/cell of wt-ad5. Ninety-six hours after infection, cells were taken for fluorescent imaging. Differentiation medium was changed every two days.
